# Four-Year Overview of Winter Colony Losses in Greece: Citizen Science Evidence That Transitioning to Organic Beekeeping Practices Reduces Colony Losses

**DOI:** 10.3390/insects14020193

**Published:** 2023-02-15

**Authors:** Evangelia Kagiali, Maria Kokoli, Philippos Vardakas, Georgios Goras, Fani Hatjina, Solenn Patalano

**Affiliations:** 1 Institute for Fundamental Biomedical Research (IFBR), Biomedical Sciences Research Center (BSRC) “Alexander Fleming”, 16672 Vari, Greece; 2 Laboratory of Sericulture and Apiculture, Department of Crop Science, School of Plant Sciences, Agricultural University of Athens, 11855 Athens, Greece; 3 Department of Cell Biology and Biophysics, Faculty of Biology, University of Athens, Panepistimiopolis, 15701 Athens, Greece; 4 Department of Apiculture, Institute of Animal Science ELGO ‘DIMITRA’, 11145 Nea Moudania, Greece

**Keywords:** COLOSS, monitoring, honey bee, forage, varroa control, survey, natural methods

## Abstract

**Simple Summary:**

Today, the monitoring and analysis of honey bee losses in more than 36 countries is carried out, in particular, through citizen science and the annual participation of volunteer beekeepers in the “COLOSS” (honey bee research non-governmental association) questionnaire on winter colony losses. Greece’s first participation in the COLOSS survey since 2018 has offered valuable analyzed data, from almost all regions, in detail for the first time in this study. In line with EU recommendations, our four-year monitoring showed a recent transition in the local beekeeping sector towards more natural practices concomitant with a decrease in overwintering colony losses. Among the different factors that influenced the winter colony losses, our study showed that the avoidance of agricultural habitats for honey production, together with the gradual substitution of chemical acaricides by more natural substances, are two major factors that could have improved colony survival. The current analysis can provide beekeepers with the relevant means to compare with their own hives’ performance. It could also serve as a basis for future more in-depth analyses of Greek winter losses and highlight beekeeping trends for the development of a successful organic transition.

**Abstract:**

The honey bee is one of the most important pollinators with a close relationship to humans. The questionnaire from the non-governmental association “COLOSS”, answered by beekeepers around the world, is a valuable tool for monitoring and analyzing factors involved in overwintering losses, as well as for understanding the evolution of the beekeeping sector over the years. Between 2018–2021, Greece’s participation in this survey involved collecting data from 752 beekeepers and 81,903 hives, from almost the whole country, with a stable balance between professional/non-professional participants and hives, providing a solid representation of the beekeeping practices and winter losses during this period. The results of this study identify a transition towards more natural beekeeping practices concomitant with a significant decrease in winter losses (average losses in 2018: 22.3% and 2019: 24%, dropped in 2020: 14.4% and 2021: 15.3%). Indeed, some factors, such as the increased use of natural landscapes for honey production (from 66.7% usage in 2018 to 76.3% in 2021) and the reduction in the exclusive use of synthetic acaricides (from 24.7% usage in 2018 to 6.7% in 2021) seem to have a significant impact on hive survival. Although these correlations remain to be confirmed experimentally, our study shows that Greek beekeepers follow recommendations and policies toward more sustainable practices. In the future, these trends could be further analyzed and integrated into training programs to strengthen the cooperation and information exchange between citizens and science.

## 1. Introduction

The honey bee is one of the most important pollinating organisms of both wild and agricultural plants [1,2,3,4,5,6,7], being essential for ecosystems’ health [6,8] and food security [2,7,9]. At the same time, it is the only pollinator with such a close relationship to humans. It is valuable for the livelihood of beekeepers [10,11,12,13], the growth of the countryside [14], and the overall global economy [2,7,15,16,17]. Since the early 2000s, increased honey bee colony losses have been observed in individual parts of the world [18,19,20,21,22,23,24,25] and universally [1,2,3]. Through the years, elevated loss rates have been attributed to different biotic and abiotic factors [18,26,27,28], such as pests and diseases [29,30,31,32,33,34] or monoculture and quality of diet [35]. In addition, the synergistic effect of many pressures is also considered to add up to an even more significant threat, compared to each individual stressor [36].

In an effort to address bee issues, as well as the compartmentation of existing research, in 2008, the international scientific research association “COLOSS” (Prevention of COlony LOSSes) was founded, to connect honey bee experts, reinforce their scientific collaboration and improve the well-being of honey bees on a global scale [18,37]. One of its core activities since the very start [38] has been the Citizen Science “Colony Losses Monitoring” project [39], which is currently active in many countries from Europe and the rest of the world [40,41,42]. The objective of this group effort is to collect and report internationally comparable data [43] concerning colony mortality during winter—the most critical season for colony losses for most European countries [44]. This is achieved through a standardized questionnaire and specific research protocol [45] that facilitate comparison between different countries, in order to identify factors with positive or negative effects on colony survival and reassess existing beekeeping practices to prevent future losses [46]. Previous analysis of COLOSS questionnaires, both at multi-country [40,41,42,44,47,48,49] and regional level [50,51,52,53,54], have set a basis for reference loss rates while investigating a combination of stressors and hive-management factors.

In Greece, only two reports dealt with winter losses between 2006–2008 and 2012–2013, but offer almost no information on beekeeping environment and practices [31,55]. However, no other record of winter losses including environmental or handling parameters was found, until the country’s first participation in the COLOSS survey in 2018 [42]. Thus, this paper aims to summarize the results of four concurrent years (2018–2021) based on the COLOSS survey, to analyze the latest beekeeping tendencies and examine the possible correlation between the colonies’ environment, beekeeping practices, and winter losses, specifically for Greece.

## 2. Materials and Methods

### 2.1. Data Collection

Global, standardized beekeeper questionnaires were translated into Greek, according to each year’s COLOSS template and requirements. Necessary adjustments to selected questions were made, following the standard COLOSS methodology [45]. Questionnaires in Greek (Supplementary File S1) were printed in paper form and published through Limesurvey’s platform (made available by COLOSS to all participating countries), during the spring of 2018–2021. An open call for beekeepers to participate voluntarily was made through our official newsletter, social media, phone communication with local beekeeping centers, and in person. A sample of 752 beekeepers was obtained directly through Limesurvey and paper-submitted surveys, which were then transferred to Limesurvey by the national monitoring team’s members. Access to the surveys was closed every July and .csv files were exported directly from Limesurvey.

### 2.2. Data Cleaning

Files in .csv format exported from Limesurvey were merged in a collective .csv file. Variables relevant to Greece only were coded in a similar pattern. All beekeepers’ personal data, date, time, and blanks were removed or filled with N/A. Regarding the question “number of apiaries”, a large number of beekeepers reported equal or more apiaries than the original colonies, or too many apiaries for the given colonies, indicating that they probably did not understand well the meaning of the question. Since none of these answers could be cross-validated, all data related to the apiaries were removed. However, beekeeper responses with problems only in this question were kept for further analysis of the rest of the variables. Only the beekeepers with more apiaries than colonies were excluded entirely, as they were considered not to understand the basic language of the survey and thus their answers were unreliable. Answers with total colonies before winter equal to N/A or zero or less than the total colony losses were also excluded. Colony migration distances, differently reported between 2019–2021, were modified to facilitate comparison. The kilometer (km) range per transportation of 2019–2020 was replaced by the km mean, and the total transportation’ km of 2021 were divided by the migration times. Migration distances of 2018 (the first year of Greek participation) were replaced with N/A as they did not include a number or distance, possibly due to problems with the Greek translation at that time. Specific Greek questions related to acaricide treatments were merged under a compatible category from the existing global variables. Answers about sugar supplement feeding reporting more than 25 kg of dry sugar per hive were also replaced with N/A, as they were not deemed realistic and probably understood by the beekeepers as kg per apiary. Greek characters or free-form text were substituted with Latin, numeric, or N/A answers depending on their translation, sometimes one by one.

### 2.3. Data Analysis

#### 2.3.1. Regional Calculations

**Figure 1:**
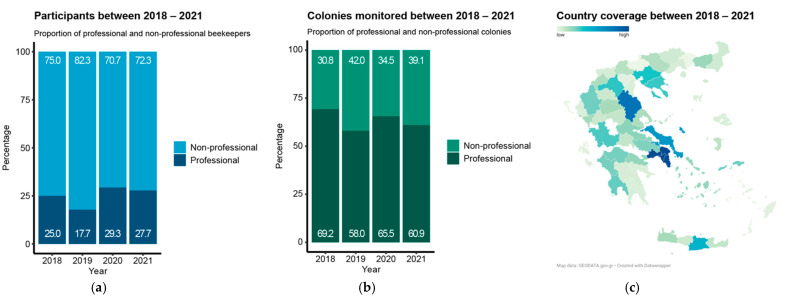
Constant distribution of the types of participants and monitored colonies across the years all over Greece: (**a**) stack bar plots showing the proportion of participants and; (**b**) monitored colonies in relation to their socio-professional situation (professional beekeepers > 150 hives); (**c**) map coverage of total participation across four years.

**Figure 2:**
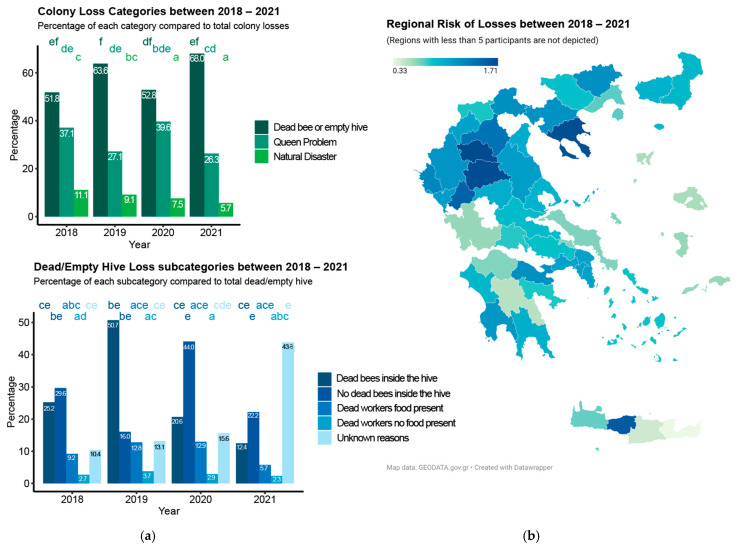
Main reasons for the loss of colonies and identification of regions at risk in Greece: (**a**) bar plots showing percentage of loss per main category (upper panel) and per sub-category (lower panel) per year; (**b**) map showing the risk of losses over the four years (the map shows beekeeper answers only for regions with a minimum of five answers over the four years). The statistical significance of the changes observed within the four years was performed using a generalized linear model with a negative binomial distribution. Multiple comparisons were performed using Tukey tests. Letters on top of each bar indicate the significance of the comparisons. When two bars share the same letter, their differences are not significant.

To maintain anonymity, regions with fewer than three participants in a particular year were excluded from the year’s regional calculations (Supplementary Tables S1 and S2) and maps (Figure 1c and Figure 2b). Similarly, regions with fewer than five participants in the total four-year period were excluded from the total regional calculations and maps. Any data excluded for anonymity reasons were used for the overall period calculations and in the comprehensive national analysis. Resulting maps were created using the Datawrapper tool, based on R .csv exports.

#### 2.3.2. Losses

A loss rate was calculated for each beekeeper as the sum of lost colonies (due to queen problems + natural disaster + worker bee deaths) divided by the colonies before winter in a given sample [43]. At each reported loss, a 95% confidence interval was calculated. The risk of loss for each region was calculated by its loss rate divided by the loss rate of the whole country, so that if the risk is >1, then there is a higher risk of losses, while if the risk is <1, then the risk is lower compared with the overall country losses.

#### 2.3.3. Categorization

The criteria for declaring a beekeeper as professional was the possession of at least 150 productive colonies before winter. Accordingly, beekeepers with fewer than 150 productive colonies before winter were considered non-professionals. Different forage plants were grouped into two categories. Orchard trees, oil seed rape, maize, sunflower, and cotton were grouped together as agricultural forage sources, and pines/conifers, thyme, heather, and cistus were grouped as natural forage sources. For each beekeeper, a score of +1 was given to each visit declared to a natural habitat (heather, pine or conifers, thyme, and cistus) and −1 to each visit to an agricultural habitat (orchards, OSR, maize, sunflowers, cotton). A positive score indicated a tendency for the beekeeper to choose natural habitats.

The different varroa treatment methods were grouped into four major categories based on previous research categorization [56]: The “Biotechnical method” includes beekeepers exclusively using hive manipulation techniques without adding substances such as drone removal, hyperthermia, or other biotechnical methods. The “Organic acid” category includes beekeepers treating hives only with natural substances such as formic acid (short- and long-term exposure), lactic acid, oxalic acid (by strip, sublimation, or combined with glycerin), or other commercial mixtures (e.g., Hiveclean^®^, Bienenwohl^®^, Varromed^®^) and Thymol (e.g., Apiguard^®^, ApilifeVar^®^, Thymovar^®^). The “Synthetic acaricide” category was assigned to beekeepers using only chemical treatments such as Tau-Fluvalinate (e.g., Apistan^®^), Flumethrin (e.g., Bayvarol^®^, Polyvar^®^), Amitraz (by strips, e.g., Apivar ^®^, Apitraz^®^, or by fumigation or aerosol), Coumaphos (by strip, e.g., Perizin^®^ or strips, e.g., Checkmite+^®^) or any other chemical substances. The “Other method” was used to label beekeepers reporting other, unspecified methods for varroa control. Finally, the category “Multiple” was created to represent beekeepers using a combination of at least 2 of the previous categories.

#### 2.3.4. Statistics

All statistical analyses were performed with the software R v4.1.3. Unless explicitly stated in the text, the description of the variability of the data over the four years was performed by providing the standard deviations of the means for each variable studied and/or using plots. 

To compare the changes in overwinter losses within the four years and across the main and the sub-categories, we used a generalized linear model with a negative binomial distribution (to account for the overdispersion and an over-representation of zero) and adding the total number of colonies before winter as “offset” (*MASS* R-package). The comparison of the estimated marginal means was performed with Tukey’s adjustment method (*multcomp* R-package).

To explore if the type of acaricide treatment and beekeeping practice affected the likelihood of colony loss, the proportion of lost colonies compared to the proportion of colonies not lost was examined using a generalized linear model with a quasibinomial distribution to account for the overdispersion (*stats* R-package). We first assigned each beekeeper a “natural score” representing environmental habits, according to which a higher score would indicate a greater beekeeper preference for using natural areas. In the first model, we tested the influence of the natural score individually and in interaction with the socio-professional category of the beekeeper. In the second model, we tested the influence of the acaricide treatment individually and in interaction with the declarations of the type of beekeeping practiced. The explanatory variables were: Model 01: the natural score (“−4” score was excluded due to low sample size) and its interaction with the professional status of the beekeeper. Model 02: the type of acaricide treatments (“organic acids”, “synthetic acaricide” and “multiple”) and its interaction with the declaration of the beekeeper (“organic” and “conventional”). The “biotechnical methods” were excluded due to the low sample size. 

## 3. Results

### 3.1. Constant Distribution of the Types of Respondents across the Years and All over Greece

During the four years of participation, 752 valid surveys were collected, representing 81,903 colonies (Table 1). For every year, more than ¾ of the total participants were non-professionals (Figure 1a), while at the same time ⅔ of the total colonies monitored belonged to professionals (Figure 1b). Although a constant decrease in participation was observed across the years, the ratio between professionals and non-professionals was always maintained. On average, a professional beekeeper had five times more hives than a non-professional (288 ± 187, versus 51 ± 36). Data from 50 out of 52 regions of Greece were obtained through the survey (Figure 1c, Supplementary Table S1), providing a good representation of the beekeeping sector all over the country. 

### 3.2. Main Reasons for the Loss of Colonies and Identification of Regions at Risk in Greece 

Every winter, about 17% of the country’s total colonies were lost, while the loss rate per Greek beekeeper was, on average, 20% over the monitoring period (Table 1). The years 2018 and 2019 were the worst years concerning the rates of overwinter losses, with 22.3% and 24% of colonies lost, respectively, with no significant difference (Table 1, 2018 versus 2019: *p* = 0.86604), followed by a good period of two years during 2020 and 2021, with 14.4% and 15.3% of colonies lost, respectively, with no significant difference either (2020 versus 2021: *p* = 0.99851). However, the drop between these two periods was statistically significant (2019 versus 2020: *p* < 0.001). Overall, non-professionals had higher loss rates, calculated at 21.5% (95%CI 19.5–23.6) compared to professionals at 15.3% (95%CI 12.7–18.0). Between the factors of the annual colony losses, a dead bee population or empty hives were always the most common cause (57.8% ± 8% of total colonies lost) followed by colonies lost due to queen problems (33% ± 6.8%). At the same time, natural disasters were not very common, contributing to an average loss rate of 9.2% ± 2.3%. We also observed that the rates of colony loss for each main category were not significantly influenced within the period, with the exception of the rates from the category “natural disaster”, which appeared to be higher during the years 2018–2019 (Figure 2a, upper panel). Within the sub-categories of dead or empty hives, the most reported factor of losses across the four years was “dead bees inside the hive” (31.7% ± 16.5%), followed by “no dead bees inside the hive” (26.1% ± 12.1%) and “dead workers with food present” (10.6% ± 3.4%). Just a few colonies were lost due to starvation, “dead workers without food present” (3% ± 0.6%), marking this category as the rarest one. However, losses due to unknown reasons were 16.9% ± 15.3% on average. Note that in 2021, the number of hives lost for unknown reasons spiked, reaching 43.8. However, none of the rate fluctuations observed across the sub-categories of losses were significant over the four years (Figure 2a, lower panel). Across the four years, the regions with the highest risk of loss were Chalkidiki, Grebena, Arta, Trikala, and Rethymnon, all in the north, except one on the island of Crete. The lowest risks were found in Samos, Lesvos, Chios, Lasithi, Heraklion, Arkadia, and Aetolia-Acarnania, all islands, except the last two, in Peloponnese (south) and central Greece (Figure 2c).

### 3.3. Queen Replacements Reduce Colony Losses

**Figure 3:**
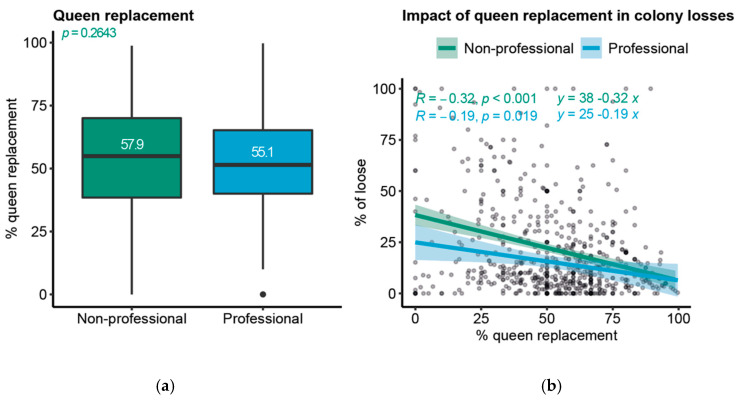
Queen replacements reduce colony losses: (**a**) box plot showing the % of queen replacement in relation to the beekeeper professional categories, *p*-value indicates the significance of unpaired two samples *t*-test; (**b**) scatter plot showing the relation between hive losses and queen replacement (*n* = 618 beekeepers, *p*-values indicate the significance of Pearson’s correlation coefficient).

Over the four-year monitoring, the country’s general rate of losses due to queen problems was 5.6%, which corresponds to the second main reason for hive losses. Beekeepers reported replacing queens in half of their hives yearly (52.9% ± 22.2%, Figure 3a), meaning that each hive had a new queen about every two years. Most regions followed the same pattern of queen replacement (Supplementary Table S2). Previous work has identified that queen replacement has a direct impact on hive losses [40,41,48]. Therefore, we investigated whether the loss rate per beekeeper correlated with their respective queen replacement rate. We found that for both professional and non-professional beekeepers, queen replacement significantly reduced colony losses (Figure 3b).

### 3.4. Increase in Use of Natural Habitats

**Figure 4:**
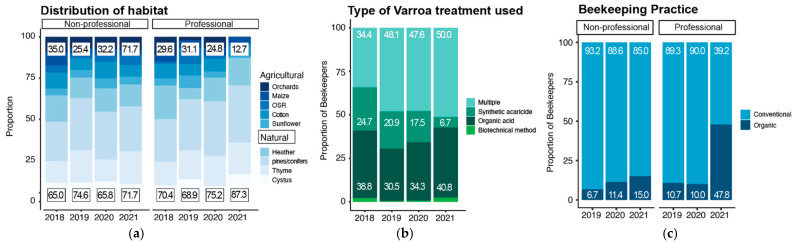
Decreases in chemical usage and evidence for a transition toward organic beekeeping: (**a**) bar plot showing the distribution of agricultural and natural plants visited for honey production with their respective percentage per year and according to their profession; (**b**) stack bar plots showing the proportion of acaricide usage across the four years; respective percentage for biotechnical method are 2018: 2.1%, 2019: 0.5%, 2020: 0.6% 2021: 2.5%. (**c**) Bar plots showing the distribution of beekeeper declarations in relation to their beekeeping practice, according to their profession, and across the years.

Beekeepers’ choice for hive wintering environments proved to be highly stable over the years. Meadows were always the preferred wintering place (38.2% ± 1.5%), followed by agricultural areas (23.4% ± 0.4%), forests (18.9% ± 3.5%), cities (6.4% ± 3.5%) or other overwintering places (13.1% ± 1.44%) (Supplementary Table S3). On average, beekeepers moved their hives about 3.2 ± 1.8 times per year, with professionals migrating slightly more, in both frequency (4.0 ± 2.2 times versus non-professional 2.9 ± 1.5 times) and distance (114.8 km ± 110.6 km versus non-professional 94.8 km ± 92.6 km for each migration travel). Overall, beekeepers used 2/3 of natural habitats (70.4% ± 4.1%) and 1/3 of agricultural habitats (29.8% ± 4.1%) to produce their honey (Figure 4a). Interestingly, we saw a slight increase in the use of natural habitats (all categories; 2018: 66.7%, 2019: 73,2%, 2020: 70.2%, 2021: 76.3%), which is mainly explained by the decrease in the use of orchards, especially by professional beekeepers in the last year (Figure 4a).

### 3.5. Decrease in the Exclusive Use of Synthetic Acaricides and Replacement by Combined Treatments

On average, 87.4% ± 8% of beekeepers checked for varroa infestations yearly (not necessarily quantitatively), while almost all of them (95.2% ± 1.5%) treated against it, regardless of doing a check (Supplementary Table S4). Over the years, natural treatments using organic acids such as oxalic and formic acid (see methods) remained relatively stable (35% ± 4.7%). In contrast, treatments using synthetic acaricides such as Amitraz dropped (18.9% ± 7.7%, Figure 4b, Supplementary Table S4). Biotechnical methods, which did not involve treatment, seemed to be used marginally in Greece (1.5% ± 1%). Interestingly, our data also described that the combination of multiple types of acaricide treatments gained popularity (43.7% ± 7.2%), which might reflect some adaptation from beekeeper habits (Figure 4b, Supplementary Table S4). 

### 3.6. Beekeepers Are Shifting toward Organic Beekeeping Practices

On average, across the four years, half of the participants used screened bottom boards (50.4% ± 2.1%) and insulated hives (47% ± 10%) (Supplementary Table S5). The use of plastic hives and frames with non-wax foundation both seemed to be more popular practices over the years (plastic hives: 19.2% ± 7.9%, non-wax foundation: 4.5% ± 1.5%), but still remained marginal practices amongst beekeepers (Supplementary Table S5). The country’s average sugar supplementing was 4.3 kg ± 0.8% and slightly increased through the years (Supplementary Table S5). Finally, our survey analysis shows that more and more beekeepers declared practicing organic beekeeping (12.6% ± 7.9%) in both professional and non-professional categories (Figure 4c). Varroa tolerant stock use (which is another way to manage varroa naturally) was also increased (17.1% ± 7.3%) (Supplementary Table S5).

### 3.7. The Use of Natural Habitats and the Combination of Multiple Acaricide Treatments Are Significantly Associated with Reduced Colony Losses

**Figure 5:**
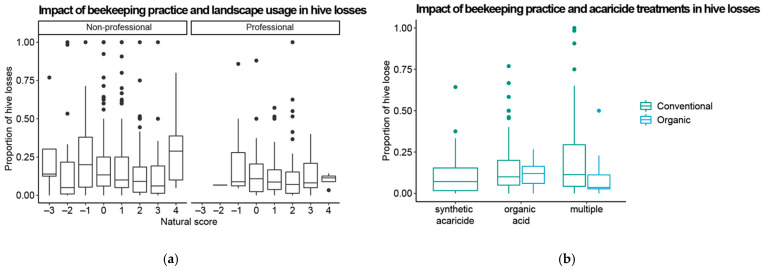
The use of natural habitats and the combination of several acaricide treatments are significantly associated with reduced colony losses: (**a**) box plots showing the variation in hive losses in relation to the natural scores obtained by beekeepers for the non-professional and professional categories; (**b**) box plots showing the variation in hive losses according to the type of acaricide treatment and the declarations of the beekeepers concerning their type of practice.

Faced with the increasing trend of organic beekeeping practices from our descriptive analysis, we explored whether the most relevant factors (increased use of natural habitats and increased use of combined acaricide treatments) influenced the results of colony losses. The results of the glm statistical analyses are shown in Table 2. The natural score alone and its interaction with the professional category significantly contributed to reduced hive losses (Figure 5a). Finally, the use of multiple types of acaricide treatments showed that this new habit of beekeepers had a beneficial effect on hive losses. However, the interaction of this practice with their declarations was not significant (Figure 5b). Taken together, our results suggest that Greek beekeepers are transitioning toward a more organic treatment of their apiaries, and such a transition could benefit colony survival.

## 4. Discussion

### 4.1. Participation

During the reference period of 2018–2021, data from 752 professional and non-professional beekeepers were collected, providing information on 81,903 hives in total. To obtain these, a mixed sampling approach was attempted, using some face-to-face interviews, internet surveys, and post or email-returned answers, based on the proposed data collection using COLOSS surveys [45]. The resulting sample, distributed all over the country and between professionals and non-professionals by a constantly maintained ratio, has provided an illustration of the country’s winter losses. However, the actual annual number of participants has been continuously declining since 2019. This is possibly due to the COVID-19 pandemic and the nationally posed restrictions on colony migrations and gatherings, which might have hindered effective communication between beekeeping centers and beekeepers. Indeed, a recent study on the effect of the COVID-19 pandemic on bee research validated its negative effect on sample collection [57]. Therefore, compared to the number of officially registered beekeepers [58,59], our sample represents only 3% of them. Even though our sample size still allows us to draw some reserved conclusions at a national level, more information is needed in order to analyze specific regional differences that are possibly present and can affect the overwintering of colonies. Hopefully, increased participation in the following years will render the opportunity to make more detailed observations in both space and time.

### 4.2. Losses

As this study focuses on winter colony losses, we need to clarify that some losses during the overwintering period are both considered normal and expected. Nevertheless, the resulting loss rates of this four-year monitoring revealed elevated losses above the 10% rate that was considered normal for the country [31]. Overall, the country’s average loss rate was 20%, ranging between 14.4% and 24%. The first two years of the analysis were characterized by high colony mortality compared to local [31] and international results [41,42] alike, while in the last two years, a significant decrease in losses was observed, compared to the initial period. With the exception of the natural disaster, the percentage of the main loss categories, such as queen problems and dead bee populations or empty hives, remained significantly stable within the four years. Similarly, the sub-categories of colony losses (dead colonies with bees inside the hive, dead colonies without dead bees inside the hive, dead colonies without food, dead with food and dead for unknown reasons) show some fluctuation but none were significant. Concerning the regional distribution of losses, the highest rates were observed in the north, with one exception (Chalkidiki, Grevena, Arta, Trikala, and Rethymnon) and the lowest in island areas and two regions in Peloponnese and central Greece (Samos, Lesvos, Chios, Lasithi, Heraklion, Arkadia, and Aetolia-Acarnania). Apart from the overall regional loss rates, no apparent geographic pattern was revealed, primarily due to insufficient data for year-to-year regional comparisons. The contrasting loss rates observed in each region in the present study have been a common phenomenon in previous studies as well [23,31,53,54,60], and apart from the factors examined here, could be influenced by the regional landscape composition [61,62,63] or specific weather conditions [64,65,66,67] and their effect on plants or pests’ life cycles [66,68,69].

### 4.3. Environment and Practices

The preferred environment for colony overwintering remained remarkably stable through the years, with meadows being selected the most and agricultural areas coming second, followed by forests, other locations, and lastly cities. On the other hand, the beekeepers’ selection of the foraging environment presented an increase towards natural sources, with bees having stable access to plants such as pines, conifers, thyme, heather, and cistus but no deliberate access to orchards for honey production. Queen replacements were conducted yearly and in a consistent manner, substituting old queens of half of the hives on an annual basis. Additionally, almost all participants were in tune with the necessary checks and treatments for varroa in their colonies. The most notable changes regarding beekeeping practices were the gradual substitution of synthetic acaricides with natural methods or substances and the increased declaration of an organic approach, especially in 2021. Such observations in our analysis have made evident that Greek beekeepers were caught in a transition to more natural environments and methods. In fact, a general tendency for organic agriculture and beekeeping has been growing in previous years, globally [70,71,72,73,74,75,76,77,78]. Only the increase in plastic hive use, which does not align with organic practices, contradicts our observed trend, but could be explained by economic reasons. The EU has already started promoting the transition to organic beekeeping through local authorities by offering economic support opportunities [79,80]. As shown in previous reports, such motives, especially in the post-pandemic world economy, have proved efficiently attractive to convince beekeepers to make the necessary changes [75,76,77,78]. Hence, based on our findings, we were able to demonstrate such a shift in the beekeeping sector. Although no intentional selection was made during the survey collection and analysis, here we still hold a small reservation as to whether younger beekeepers with access to the online form of the survey are possibly more interested in organic beekeeping practices.

### 4.4. Practices Effects on Losses

The elevated queen replacement was positively linked with reduced colony losses, in agreement with previous findings [40,41,48]. Decreased access to agricultural foraging environments and the substitution of synthetic varroa treatments were also correlated with a lower loss risk. On the contrary, declarations of organic practice did not influence winter mortality, as they most probably reflected the beekeepers’ philosophy or intentions and not the actual conditions of their hives. Through the previous bibliography, we see that foraging in agricultural environments, instead of natural ones, can negatively affect winter colony survival [42,48,53]. This can be attributed to the poorer nutrition obtained from a monoculture diet, compared to a diversity of wild plants [35,48,81], and the possible exposure to sub-lethal doses of crop chemicals [52,82,83]. Furthermore, synthetic acaricides have been blamed for building resistance, polluting the hive’s interior, and causing bee health complications [84,85]. Exposure to chemicals from varroa control treatments, in-hive residue, and intensified agriculture is also typically found to lead to more losses [52,82,83,86]. Based on the above, our findings may reinforce the opinion that queen replacements and a more natural beekeeping approach, in terms of foraging and varroa control, could be considered in the prevention of colony losses, at least locally. Still, patience is advised in order to observe the complete substitution of synthetic acaricides, as different varroa fighting strategies have their advantages and weaknesses [85]. 

### 4.5. Future Perspectives

Of course, any observational study like this one has its limitations [87,88], and alone it should be interpreted with care; supplementary research from hive samples may prove necessary in some cases to accurately assess the effects of this organic transition. A sure causative link between fewer winter losses and more natural beekeeping practices may be established after a controlled experimental study. Consequently, the present effort can only signify the association between the two. In the meantime, educational opportunities for organic beekeepers should be promoted, given the recent shift observed in the sector and the importance of improved beekeeping knowledge for colony survival [89]. Resources and scientific findings from efforts like the present study could complement these. In fact, beekeeper training programs could be designed with the integrated annual monitoring of colonies to reinforce the cooperation and information exchange between citizens and science, on the way to a better future for bees. Moreover, the investigation of organic beekeeping possibilities [70,74,90,91,92], even after the state’s economic support, would also be beneficial for Greek application in order to encourage the beekeepers’ transition, and to result in higher quality honey bee products, healthier food systems, increased sustainability standards [74,78,79] and improved colony health and survival for the benefit of bees and beekeepers alike.

## 5. Conclusions

Thanks to Greece’s four-year participation in the COLOSS survey, we were able to observe that the last two years (2020–2021) were marked by a significant decrease in winter losses, concurrent with an increase in natural beekeeping practices. In fact, the collected data outlined a clear image of transitioning to more sustainable and organic beekeeping practices, in accordance with the EU’s recommendations. While most of the factors that were analyzed (such as queen replacement, overwintering environment, and beekeeper migration practices) remained stable over this period, the increased use of natural habitats for honey production and the gradual substitution of synthetic acaricides were significantly associated with a decrease in the winter colony losses. For now, our results can certainly provide some feedback to previous participants, concerning different practices and average loss rates, which are helpful for personal comparison.

## Figures and Tables

**Table 1:** Summary of participation and hive loss rate.

Year	Type	Number ofParticipants	Number of Hives	Mean Loss Rate % (95% CI)
2018	Non-professional Professional Total	201 67 268	9326 20,964 30,290	23.9% (19.8–28.0) 17.6% (12.0–23.1) 22.3% (19.0–25.7)
2019	Non-professional Professional Total	171 37 208	8562 11,820 20,382	25.7% (22–29.3) 16.1% (10.1–22.0) 24% (20.8–27.2)
2020	Non-professional Professional Total	106 44 150	6283 11,943 18,226	14.8% (11.5–18.0) 13.4% (9.1–17.6) 14.4% (11.8–19.9)
2021	Non-professional Professional Total	91 35 126	5076 7929 13,005	16.3% (12.3–20.3) 12.8% (8.7–17.0) 15.3% (12.2–18.4)
All YEARS	Non-professional	569	29,247	21.5% (19.5–23.6)
	Professional	183	52,656	15.3% (12.7–18.0)
All YEARS	Total	752	81,903	20.0% (18.4–21.7)

**Table 2:** Results of the generalized linear models, which were performed to test the effect of natural habitat usage (Μodel 01) and acaricide treatment (Model 02) on colony losses.

Predictors	Estimate	Std. Error	*t* Value	Pr (>|*t*|)
Model 01: Loss rate ~ Natural Score + Natural Score: Professional type				
(Intercept)	1.44274	0.07035	−20.507	**<2 × 10^16^** ***
Natural Score	−0.14913	0.06356	−2.346	**0.0193** *
Natural Score: type Professional	−0.13639	0.07385	−1.847	**0.0652 .**
Model 02: Loss rate ~ Acaricide treatment + Acaricide treatment: Organic Beekeeper				
(Intercept)	−2.20059″	0.23146	−9.508	**<2 × 10^16^** ***
Varroa_category organic acid	0.38583″	0.27999	1.378	0.1693
Varroa_category multiple	0.60984	0.25115	2.428	**0.0158** *
Varroa_category synthetic acaricide: OrganicBeek	NA	NA	NA	NA
Varroa_category organic acid: Organic Beekeeper	−0.04581	0.27261	−0.168	0.8667
Varroa_category multiple: Organic Beekeeper	−0.54119	0.34488	−1.569	0.117

Bold values indicate significance (0 *** 0.01 * 0.05 .).

## Data Availability

Cleaned and anonymized raw data are available on request to patalano@fleming.gr.

## Supplementary Materials

The following supporting information can be downloaded at: https://www.mdpi.com/article/10.3390/insects14020193/s1, Table S1: Summary of participation and risk of hive losses across the years and regions, Table S2: Summary of queen replacement rates across the years and regions, Table S3: Summary of the overwintering place across the years, Table S4: Summary of Acaricide treatment across the years, Table S5: Summary of Beekeeping practices across the years, File S1: Beekeeper surveys 2018–2021.
