# Supplementary material for: Four-Year Overview of Winter Colony Losses in Greece: Citizen Science Evidence That Transitioning to Organic Beekeeping Practices Reduces Colony Losses
Source: Insects. 2023 Feb 15;14(2):193. doi: 10.3390/insects14020193 (PMC9963079; doi:10.3390/insects14020193)
Supplement: Supplementary file 1 [file insects-14-00193-s001.zip › insects-2104521-supplementary-Tables S1-S5.pdf]

Four-Year Overview of Winter Colony Losses in Greece: Citizen Science Evidence That Transitioning to Organic Beekeeping Practices Reduces Colony Losses

Supplementary Materials

To maintain anonymity, regions with fewer than three participants in a particular year were excluded from the year's regional calculations. Similarly, regions with fewer than five participants in the total reference period were also excluded from the total years' calculations (Sup. Tables S1, S2).

Table S1: Summary of participation and risk of hive losses across the years and regions

Region	2018	2019	2020	2021	Total Years
Achaias		0.66	0.77	0.97	0.66
Aitoloakarnanias	0.60	0.58	0.49	0.84	0.61
Argolidas	0.69				0.8
Arkadias					0.51
Artas	1.89				1.53
Attikis	1.13	1.32	0.57	1.08	1.08
Biotias	1.21	0.28	0.84	0.71	0.86
Chalkidikis	1.60	1.26	1.87		1.68
Chanion	1.32	0.52	0.55		0.72
Chiou	0.73		0.41		0.6
Dramas		1.01			1.21
Evoias	0.88	0.39	1.3	0.37	0.67
Evrou	0.1	1.26			0.91
Florinas		0.88			0.8
Fokidas	0.82				0.8
Fthiotidas		0.73	1.46	1.16	0.89
Grebenon	2.27				1.71
Ilias	1.39		1.14	0.22	0.91
Ioanninon	1.21	0.89			1.17
Irakleiou	0.35	0.5	0.26		0.45
Karditsas		1.62	0.63		1
Kastorias		0.8	2.54		1.08
Kavalas					0.74
Korinthias	1.43	0.83	1.01	1.8	1.21
Kozanis	1.08	1.71	1.01	0.97	1.42
Kykladon	0.72	1.01	0.58	1.01	0.85
Lakonias			1.7		0.98
Larisas	1.15	1.09	0.26	0.73	1.1
Lasithiou	0.27				0.33
Lesbou					0.56

Magnisias	0.14	1.93	0.37	1.77	0.95
Messinias	0.88	1.38	1.14		1.15
Pellas	1.43		0.29		1.22
Pierias					1.01
Prevezas	0.84	1.11	1.89		1.1
Rethymnis		1.88			1.6
Rodopis	0.94				0.96
Samou		0.52	0.72	0.83	0.61
Serron	0.67	0.98			0.85
Thesprotias	1.52				1.28
Thessalonikis	1.28	0.72	1.27	1.96	1.19
Trikalon	0.66	1.74		3.36	1.69
Zakynthou			0.25		

Table S2. Summary of queen replacement rates across the years and regions

Region	2018	2019	2020	2021	Total Years
Achaias		61.50%	67.10%	61.20%	65.90%
Aitoloakarnanias	60.20%	58.80%	57.50%	29.50%	53.40%
Argolidas	69%				65.50%
Arkadias					53.90%
Artas	27%				41.80%
Attikis	48.30%	49%	62.90%	58.40%	54.20%
Biotias	57.20%	70.90%	64.10%	62.90%	62.80%
Chalkidikis	77%	58.40%	70.20%		70.20%
Chanion	75.60%	59.10%	38.30%		55.10%
Chiou	77.60%		59.30%		62.40%
Dramas		20.40%			37.60%
Evoias	89.50%	46.80%	61%	59.40%	61.20%
Evrou	85.90%	40.30%			59.80%
Florinas		62.80%			66.90%
Fokidas	36.20%				55.40%
Fthiotidas		74%	50.70%	43.90%	59.40%
Grebenon	67.60%				47.40%
Ilias	88.80%		45.60%	39.60%	55.90%
Ioanninon	55.60%	61.70%			56%
Irakleiou	62.90%	49.90%	75.70%		58.40%
Karditsas		32.30%	51.40%		46.70%
Kastorias		87.20%	119%		95.10%
Kavalas					57.30%
Korinthias	52.80%	66.70%	56.40%	47.60%	55.20%
Kozanis	76%	36.80%	49.70%	47.20%	49.50%
Kykladon	65.20%	61.40%	70.60%	43.90%	60.10%
Lakonias			53.60%		59.80%
Larisas	73.30%	53.60%	65.70%	39.20%	61.30%

Lasithiou	71.20%				68.80%
Lesbou					46.40%
Magnisias	58.90%	27.50%	62.90%	52.80%	51.20%
Messinias	63.90%	63.60%	48.10%		60.50%
Pellas	53.40%		55.70%		54.20%
Pierias					49.50%
Prevezas	59.20%	44.40%	54.30%		54.70%
Rethymnis		48.20%			38%
Rodopis	62.70%				52.10%
Samou		50.30%	77.20%	55.10%	54.70%
Serron	45.30%	54.90%			50.40%
Thesprotias	112.50%				84.70%
Thessalonikis	67.90%	62.80%	55.30%	47.50%	59.40%
Trikalon	79.20%	32.10%		19.20%	40.90%
Zakynthou					

Table S3: Summary of overwintering locations across the years

Location	2019	2020	2021	Total Years (%±SD)
City	3.9%	10.7%	5.6%	6.4% ±3.5%
Forest	20.9%	14.7%	20.6%	18.9% ±3.5%
Meadow	37.4%	40%	37.3%	38.2% ±1.5%
Agricultural area	23.8%	23.3%	23%	23.4% ±0.4%
None of the above	14.1%	11.3%	13.5%	13.1% ±1.44%

Table S4. Summary of Acaricide treatment across the years

Method	2018	2019	2020	2021	Total Years (%±SD)
Checking varroa	94 %	77.3%	84.6 %	93.7 %	87.4% ±8%
Apply treatments	93.6 %	95.2 %	96.6 %	96.8 %	95.2% ±1.5%
Biotechnical	2.20%	0.53%	0.70%	2.50%	1.5% ±1%
Synthetic	24.67%	20.86%	17.48%	6.67%	18.9% ±7.7%
Organic acid	38.77%	30.48%	34.27%	40.83%	35% ±4.7%
Multiple	34.36%	48.13%	47.55%	50%	43.7% ±7.2%

Table S5: Summary of beekeeper practices across the years

Practice	2019	2020	2021	Total Years
Use screened bottom boards	48.8 %	52.8 %	50 %	50.4% ±2.1%
Use insulated hives	45 %	39.3 %	58.8 %	47% ±10%
Use plastic hives	13 %	19.8 %	28.7 %	19.2% ±7.9%
Use non-wax foundation	5.9 %	3 %	3.8 %	4.5% ±1.5%
Sugar supplementing	4.1 kg	3.7 kg	5.3 kg	4.3 kg ±0.8kg
Use varroa tolerant stock	15.1%	12.5%	26.2%	17.1% ±7.3%
Do organic beekeeping	7.4%	10.8%	24.1%	12.6% ±7.3%
