# Supplementary material for: Four-Year Overview of Winter Colony Losses in Greece: Citizen Science Evidence That Transitioning to Organic Beekeeping Practices Reduces Colony Losses
Source: Insects. 2023 Feb 15;14(2):193. doi: 10.3390/insects14020193 (PMC9963079; doi:10.3390/insects14020193)
Supplement: Supplementary file 1 [file insects-14-00193-s001.zip › insects-2104521-Supplementay File S1-Beekeeper surveys 2018-2021.pdf]

www.coloss.org

Ερωτηματολόγιο για τις απώλειες των μελισσών 2017-2018

Αποστείλετε αυτό το ερωτηματολόγιο στο e-mail: beemonitorgr@gmail.com

Έως τις 31 Μαΐου 2018

Για περισσότερες πληροφορίες επικοινωνήστε μαζί μας! Monitoring Greece- Beekeepers Survey

ΠΡΟΣΩΠΙΚΕΣ ΠΛΗΡΟΦΟΡΙΕΣ

Μη σημπληρώνετε εαν θέλετε ανώνυμη απάντηση, σε περίπτωση που θα τις συμπληρώσετε τα στοιχεία σας θα αφαιρεθούν και θα παραμείνουν εμπιστευτικά.

Όνομα	______________________________
Επίθετο	______________________________
Διεύθυνση	______________________________
Πόλη	______________________________
ΤΚ	______________________________
Email	______________________________

Οι ερωτήσεις σε κύκλο (1, 2, 3...) πρέπει οπωσδήποτε να απαντηθούν
Οι ερωτήσεις σε τετράγωνο (10, 11, 12...)είναι προαιρετικές

ΤΟΠΟΣ

1 Σε ποιο νομό βρισκόταν η πλειοψηφία των μελισσοκομείων (ομάδων κυψελών) σας κατά τη διάρκεια του **χειμώνα*** του 2017-18; **ΝΟΜΟΣ:** ______________

***Χειμώνας θεωρείται η περίοδος μετά τις τελευταίες προετοιμασίες για το κρύο μέχρι την έναρξη της νέας περιόδου σίτισης**

ΜΕΛΙΣΣΟΚΟΜΕΙΑ (ΟΜΑΔΕΣ ΚΥΨΕΛΩΝ)

2 Πόσα μελισσοκομεία (ομάδες κυψελών)έχετε; **ΑΡΙΘΜΟΣ:** ☐

3 Εάν έχετε πάνω από ένα μελισσοκομείο, η απόσταση μεταξύ όλων των μελισσοκομείων σας είναι μέχρι 15 χλμ; Εάν έχετε μόνο ενα μελισσοκομείο, παρακαλώ να απαντήσετε Ναι.

○ **Ναι** ○ **Όχι** ○ **Δεν ξέρω**

ΠΑΡΑΓΩΓΙΚΑ ΜΕΛΙΣΣΙΑ

4 Πόσα **παραγωγικά μελίσσια*** είχατε πριν το χειμώνα του 2017-2018; **ΑΡΙΘΜΟΣ:** ☐

***Παραγωγικά μελίσσια θεωρούνται τα μελίσσια που είναι αρκετά υγιή ώστε να δώσουν παραγωγή μελιού.**

ΑΠΩΛΕΙΕΣ ΤΩΝ ΜΕΛΙΣΣΩΝ

Από τα **παραγωγικά σας μελίσσια**:

5 Πόσα μελίσσια με σοβαρά **προβλήματα βασιλισσών επέζησαν** κατά τη διάρκεια του χειμώνα; (Πολλοί κηφήνες ή εξαφάνιση βασίλισσας). Εάν δεν υπήρχαν προβλήματα παρακαλούμε να απαντήσετε **0**. **ΑΡΙΘΜΟΣ :** ☐

6 Πόσα μελίσσια **χάσατε**, λόγω **φυσικών καταστροφών**; (Χιόνια, πλημμύρες, αρκούδες, κλοπές, άλλα…) **ΑΡΙΘΜΟΣ :** ☐

7 Πόσα μελίσσια **χάσατε**, λόγω **θανάτων μελισσών**; **ΑΡΙΘΜΟΣ :** ☐

8 Πόσα από τα νεκρά μελίσσια ή τις άδειες κυψέλες είχαν:

α)**Πολλές νεκρές μέλισσες** μέσα ή μπροστά από την κυψέλη **ΑΡΙΘΜΟΣ :** ______

β)**Λίγες ή καθόλου νεκρές μέλισσες** μέσα ή μπροστά από την κενή κυψέλη **ΑΡΙΘΜΟΣ :** ______

γ)νεκρές εργάτριες μέσα στα κελιά, χωρίς τροφή **(πείνα)** **ΑΡΙΘΜΟΣ :** ______

δ)**Νεκρές εργάτριες μέσα στα κελιά** αλλά **με επαρκή τροφή** στην κυψέλη **ΑΡΙΘΜΟΣ :** ______

ε)κανένα από τα παραπάνω ή **άγνωστες αιτίες** **ΑΡΙΘΜΟΣ :** ______

9 Πόσα μελίσσια **επέζησαν**, ήταν **αδύναμα αλλά είχαν βασίλισσα μετά το χειμώνα;** ☐

ΕΞΈΛΙΞΗ ΤΩΝ ΜΕΛΙΣΣΩΝ

10 Πόσα παραγωγικά μελίσσια είχατε την α) **άνοιξη*** του 2017 (πέρυσι); ______

*__Άνοιξη :__ αρχή της περιόδου συλλογής τροφής β) άνοιξη του 2018 (φέτος); ______

11 Πόσα από τα μελίσσια που επέζησαν είχαν καινούργια βασίλισσα το 2017;

ΑΡΙΘΜΟΣ :______ ☐ **Δεν ξέρω**

12 Τα προβλήματα της βασίλισσας που παρατηρήσατε φέτος κατά την περίοδο συλλογής τροφής σε σχέση με αυτά που συνήθως έχετε, ήταν:

◯**Περισσότερα** ◯**Τα ίδια** ◯**Λιγότερα** ◯**Δεν ξέρω**

13 Κατά τη διάρκεια του χειμώνα, τα μελίσσια με τις παλιές βασίλισσες σε σύγκριση με τα μελίσσια με τις νέες βασίλισσες επέζησαν:

◯**Καλύτερα** ◯**Το ίδιο** ◯**Χειρότερα** ◯**Δεν ξέρω**

14 Πόσα από τα μελίσσια που επέζησαν είχαν μεγάλη ποσότητα περιττωμάτων μέσα στην κυψέλη;

ΑΡΙΘΜΟΣ :______ ☐ **Δεν ξέρω**

ΜΕΤΑΚΙΝΗΣΗ ΚΑΙ ΠΕΡΙΒΑΛΛΟΝ ΤΩΝ ΜΕΛΙΣΣΩΝ

15 Μετακινήσατε κάποιο μελίσσι σας τουλάχιστον μία φορά για παραγωγή μελιού ή επικονίαση το 2017; ◯**Ναι** ◯**Όχι** ◯**Δεν ξέρω**

21 Εάν Ναι:

α)Ποιος ήταν ο μέσος αριθμός μετακινήσεων ανά μελίσσι το 2017;

◯**1** ◯**2** ◯**3** ◯**Άλλα** :______

β)Ποια ήταν η μέση απόσταση των μετακινήσεων ;

◯ **15-50 χλμ** ◯**50-100 χλμ** ◯**100-250 χλμ** ◯ **250-500 χλμ** ◯ **>500 χλμ**

17 Είχε η πλειοψηφεία των μελισσιών σας σημαντική παρουσία σε ένα ή περισσότερα από τα παρακάτω φυτά; Παρακαλούμε επιλέξτε μία απάντηση για κάθε φυτό:

α) **Εσπεριδοειδή** ◯Ναι ◯Όχι ◯Δεν ξέρω
β) **Ελαιοκράμβη** ◯Ναι ◯Όχι ◯Δεν ξέρω
γ) **Καλαμπόκι** ◯Ναι ◯Όχι ◯Δεν ξέρω
δ) **Ηλιοτρόπιο** ◯Ναι ◯Όχι ◯Δεν ξέρω
ε) **Ερείκη** ◯Ναι ◯Όχι ◯Δεν ξέρω
στ) **Πεύκο ή Έλατο** ◯Ναι ◯Όχι ◯Δεν ξέρω
ζ) **Θυμάρι** ◯Ναι ◯Όχι ◯Δεν ξέρω
η) **Βαμβάκι** ◯Ναι ◯Όχι ◯Δεν ξέρω
θ)**Λαδανιά** ◯Ναι ◯Όχι ◯Δεν ξέρω
ι) **Άλλα** : ______

ΒΑΡΡΟΑ

18 Παρακολουθήσατε τα μελίσσια σας για το βαρρόα κατά την περίοδο από τον Απρίλη του 2017 μέχρι τον Απρίλη του 2018; ◯**Ναι** ◯**Όχι** ◯**Δεν ξέρω**

19 Υποβάλατε σε θεραπεία τα μελίσσια σας κατά του βαρρόα κατά την περίοδο από τον Απρίλη του 2017 μέχρι τον Απρίλη του 2018; ◯**Ναι** ◯**Όχι** ◯**Δεν ξέρω**

20 Παρακαλούμε να σημειώσετε τους μήνες, κατά τους οποίους παρακολουθήσατε τα παραγωγικά σας μελίσσια για το βαρρόα ΚΑΙ σημειώστε επίσης πότε ΞΕΚΙΝΗΣΑΤΕ θεραπεία ή διαχείρηση για το βαρρόα κατά την περίοδο από τον Απρίλη του 2017 μέχρι τον Απρίλη του 2018	**2017**									**2018**			
	**Απρίλιος**	**Μάιος**	**Ιούνιος**	**Ιούλιος**	**Αύγουστος**	**Σεπτέμβριος**	**Οκτώβριος**	**Νοέμβριος**	**Δεκέμβριος**	**Ιανουάριος**	**Φεβρουάριος**	**Μάρτιος**	**Απρίλιος**
Παρακολούθηση του επιπέδου προσβολής από το βαρρόα													
Αφαίρεση κηφηνογόνου													
Υπερθερμία (Θεραπεία υπερθέρμανσης του γόνου/των μελισσών)													
Άλλες τεχνικές (όπως π. Χ. Ολική αφαίρεση γόνου, περιορισμός βασίλισσας, κλπ)													
Φορμικό Οξύ - βραχυπρόθεσμα													
Φορμικό Οξύ - μακροπρόθεσμα													
Γαλακτικό οξύ													
Οξαλικό οξύ - διαβροχή													
Οξαλικό οξύ - ψεκασμός - εξάχνωση (Hiveclean/Bienenwohl/Varromed)													
Θυμόλη (π.χ. Apiguard, ApilifeVar)													
Tau-fluvalinate (π.χ. Apistan)													
Φλουμεθρίνη (e.g. Bayvarol)													
Amitraz σε ταινιές													
Amitraz (υποκαπνισμός/αεροζόλ)													
Coumaphos (π.χ. Perizin)													
Coumaphos (σε ταινίες π.χ. Checkmite+)													
Άλλο χημικό προϊόν													
Άλλη μέθοδος													

Έχετε κάτι άλλο να μας πείτε;

____
____
____
____

Σας ευχαριστούμε πολύ για τη συμμετοχή σας!

Αποθηκεύσετε και αποστείλετε στο e-mail: beemonitorgr@gmail.com

ΠΛΗΡΟΦΟΡΙΕΣ – ΟΔΗΓΙΕΣ ΓΙΑ ΤΟ ΕΡΩΤΗΜΑΤΟΛΟΓΙΟ ΑΥΤΟ

Τι είναι το COLOSS;

Είναι μία Διεθνής Επιστημονική Ένωση που αποτελείται από 1019 μέλη από 95 χώρες που έχει στόχο την βελτίωση των συνθηκών ζωής των μελισσών.

Ποιοι είμαστε εμείς;

Είμαστε ερευνητές/ μέλη του COLOSS που συμμετέχουμε σε μία έρευνα για την παρακολούθηση απωλειών μελισσών στην Ελλάδα και το πως αυτές οι απώλειες σχετίζονται με παράγοντες όπως πχ το βαρρόα. Αντίστοιχη έρευνα γίνεται και σε πολλές άλλες χώρες.

Τι μπορείτε να κάνετε;

Η παρακολούθηση των μελισσών γίνεται μέσω ενός ερωτηματολογίου που για να συμπληρωθεί απαιτούνται μόνο λίγα λεπτά. Θα σας παρακαλούσαμε να το συμπληρώσετε. Το ερωτηματολόγιο, αν το επιθυμείτε, είναι τελείως ανώνυμο.

Τι περιέχει το ερωτηματολόγιο ;

Η βασική ερώτηση είναι πόσα μελίσσια χάθηκαν μέσα στον τελευταίο χειμώνα. Επιπλέον έχει κάποιες ερωτήσεις που αφορούν τις αιτίες των απωλειών των μελισσιών.

Γιατί να ενδιαφερθώ ;

Αν είστε μελισσοκόμος χρόνια γνωρίζετε ήδη αρκετά πράγματα και θεωρείστε επαγγελματίας. Ερευνητές σε παγκόσμια κλίμακα αφιέρωσαν χρόνο για να ανακαλύψουν αυτά που σήμερα θεωρούμε δεδομένα. Υπάρχει περιθώριο για μεγάλη ακόμα πρόοδο στο πεδίο της μελισσοκομίας. Η έρευνα χρειάζεται τους μελισσοκόμους ώστε να δείξουν τον δρόμο και τα προβλήματα που υπάρχουν.

Ελληνική Ομάδα παρακολούθησης Απωλειών Μελισσιών

Παναγίδης Νίκος COLOSS Συντονιστής, Φοιτητής Γεωπονικό Πανεπιστήμιο Αθηνών

Σολενν Παταλανο, Δρ. Βιολογός, Ινστιτούτο Φλέμινγκ, Βάρη

Λετίσια Παπουτσή Υποψήφια Διδάκτωρ Εργαστήριο Γεωργικής Ζωολογίας & Εντομολογίας Γεωπονικό Πανεπιστήμιο Αθηνών

Βαΐα Βουκάτα Φοιτήτρια Γεωπονικό Πανεπιστήμιο Αθηνών

Φανή Χατζήνα Δρ. Βιολόγος, Ερευνήτρια Α΄ Τμήμα Μελισσοκομίας ΕΛΓΟ «ΔΗΜΗΤΡΑ»

Μαρία Μπουγά Δρ. Βιολόγος, Εξωτερικός Επιστημονικός Συνεργάτης Εργαστήριο Γεωργικής Ζωολογίας & Εντομολογίας Γεωπονικό Πανεπιστήμιο Αθηνών

www.coloss.org

Ερωτηματολόγιο για τις απώλειες των μελισσών 2018-2019

Αποστείλετε αυτό το ερωτηματολόγιο στο e-mail: beemonitorgr@gmail.com

Έως τις 31 ΙΟΥΛΙΟΥ 2019

Για περισσότερες πληροφορίες επικοινωνήστε μαζί μας! Monitoring Greece - Beekeepers

ΠΡΟΣΩΠΙΚΕΣ ΠΛΗΡΟΦΟΡΙΕΣ

Οι πληροφορίες ονόματος και διεύθυνσης δεν είναι υποχρεωτικές. Εάν τις δώσετε, θα κρατηθούν με μυστικότητα και δεν παρέχονται σε τρίτους για την προστασία του απορρήτου σας.

.

Όνομα	______________________
Επίθετο	______________________
Διεύθυνση	______________________
Πόλη	______________________
ΤΚ	______________________
Email	______________________

Οι ερωτήσεις σε κύκλο (1, 2, 3...) είναι υποχρεωτικές

Οι ερωτήσεις σε Τετράγωνο (10, 11, 12...) είναι προαιρετικές

ΤΟΠΟΣ

1 Για να γνωρίζουμε καλύτερα σε ποια περιοχή ήταν τα περισσότερα μελίσσια σας τον χειμώνα* που πέρασε, πέστε μας

α) Ποιος είναι ο ΝΟΜΟΣ που βρίσκεται το κυρίως μελισσοκομείο σας ; ______________

β) Το όνομα της κοντινότερης πόλης ________________

γ) Τι είδος περιοχής ήταν; ◯ **Δάσος** ◯ **Λιβάδι** ◯ **Πόλη** ◯ **Άλλο**:________________

*<u>Χειμώνας</u> θεωρείται η περίοδος μετά τις τελευταίες προετοιμασίες για το κρύο μέχρι την έναρξη της νέας περιόδου συλλογής τροφής

ΜΕΛΙΣΣΟΚΟΜΕΙΑ (ΟΜΑΔΕΣ ΚΥΨΕΛΩΝ)

2 Πόσα μελισσοκομεία έχετε; **ΑΡΙΘΜΟΣ**: ________

3 Εάν έχετε πάνω από ένα μελισσοκομείο, είναι η απόσταση μεταξύ όλων των μελισσοκομείων σας μέχρι 15 χλμ; Εάν έχετε μόνο ένα μελισσοκομείο, παρακαλώ να απαντήσετε Ναι.

◯ **Ναι** ◯ **Όχι** ◯ **Δεν γνωρίζω**

ΠΑΡΑΓΩΓΙΚΑ ΜΕΛΙΣΣΙΑ

❹ Πόσα **παραγωγικά μελίσσια*** είχατε πριν το χειμώνα του 2018-2019; **ΑΡΙΘΜΟΣ:** ________

***Παραγωγικά μελίσσια θεωρούνται τα μελίσσια που είναι αρκετά υγιή για να θεωρήσετε ότι θα ξεχειμωνιάσουν**

Στις παρακάτω ερωτήσεις πρέπει να απαντήσετε για τις απώλειες των μελισσιών σας.
Θεωρείστε ΑΠΩΛΕΙΑ ένα μελίσσι που :
α) είναι ζωντανό αλλά έχει σοβαρά προβλήματα βασίλισσας (π.χ. έχει χαθεί η βασίλισσα ή είναι αρρενοτόκο ή δεν μπορέσατε να κάνετε κάτι)
β) χάθηκε λόγω φυσικών καταστροφών (Χιόνια, πλημμύρες, αρκούδες, κλοπές)
γ) ήταν ολόκληρο νεκρό ή έχει μείνει με μερικές εκατοντάδες μέλισσες μόνο.

ΑΠΩΛΕΙΕΣ ΜΕΛΙΣΣΙΩΝ

Από τα **παραγωγικά σας μελίσσια**:

❺ Πόσα μελίσσια **χάθηκαν** επειδή είχαν σοβαρά **προβλήματα βασιλισσών** κατά τη διάρκεια του **χειμώνα;** (π.χ. έχει χαθεί η βασίλισσα ή είναι αρρενοτόκο ή δεν μπορέσατε να κάνετε κάτι).
Εάν δεν υπήρχαν προβλήματα παρακαλούμε να απαντήσετε **0**. **ΑΡΙΘΜΟΣ :** ________

❻ Πόσα μελίσσια **χάθηκαν**, λόγω **φυσικών καταστροφών**; (Χιόνια, πλημμύρες, αρκούδες, κλοπές άλλα…) **ΑΡΙΘΜΟΣ :** ________

❼ Πόσα μελίσσια **χάθηκαν**, λόγω **θανάτων μελισσών**;
(ήταν ολόκληρο νεκρό ή έχει μείνει με μερικές εκατοντάδες μέλισσες) **ΑΡΙΘΜΟΣ :** ________

8 Πόσα από τα νεκρά μελίσσια (από αυτά που απαντήσατε στην Ερώτηση 7) ή τις άδειες κυψέλες είχαν:

α)**Πολλές νεκρές μέλισσες** μέσα ή μπροστά από την κυψέλη **ΑΡΙΘΜΟΣ :** ________
β)**Λίγες ή καθόλου νεκρές μέλισσες** μέσα ή μπροστά από την κενή κυψέλη **ΑΡΙΘΜΟΣ :** ________
γ) Νεκρές εργάτριες μέσα στα κελιά **από πείνα** **ΑΡΙΘΜΟΣ :** ________
δ) **Νεκρές εργάτριες μέσα στα κελιά αλλά με επαρκή τροφή** στην κυψέλη **ΑΡΙΘΜΟΣ :** ________
ε) κανένα από τα παραπάνω ή **άγνωστες αιτίες** **ΑΡΙΘΜΟΣ :** ________

❾ Πόσα μελίσσια από αυτά που **επέζησαν (είχαν βασίλισσα),** ήταν **αδύναμα μετά το χειμώνα;**

ΑΡΘΙΜΟΣ ________ ☐ **Δεν γνωρίζω**

ΕΞΕΛΙΞΗ ΤΩΝ ΜΕΛΙΣΣΩΝ

10 Πόσα παραγωγικά μελίσσια είχατε την α) **άνοιξη*** του 2018 (πέρυσι); ________
β) άνοιξη του 2019 (φέτος); ________

***Άνοιξη** : αρχή της περιόδου συλλογής τροφής

⓫ Πόσα από τα μελίσσια που επέζησαν τον χειμώνα είχαν νέα βασίλισσα ? (του 2018);

ΑΡΙΘΜΟΣ :________ ☐ **Δεν γνωρίζω**

12 Τυχόν προβλήματα στις βασίλισσας που παρατηρήσατε τον άνοιξη και το καλοκαίρι του 2018 ήταν συγκριτικά με αυτά που συνήθως έχετε,

○ **Περισσότερα** ○ **Τα ίδια** ○ **Λιγότερα** ○ **Δεν γνωρίζω**

13 Κατά τη διάρκεια του χειμώνα, τα μελίσσια με τις παλιές βασίλισσες (του 2017) σε σύγκριση με τα μελίσσια με τις νέες βασίλισσες (του 2018) επέζησαν:

○ **Καλύτερα** ○ **Το ίδιο** ○ **Χειρότερα** ○ **Δεν γνωρίζω**

14 Πόσα από τα μελίσσια που <u>επέζησαν</u> είχαν μεγάλες ποσότητες περιττωμάτων μέσα στην κυψέλη;

ΑΡΙΘΜΟΣ :________ ☐ **Δεν γνωρίζω**

ΜΕΤΑΚΙΝΗΣΗ ΚΑΙ ΠΕΡΙΒΑΛΛΟΝ ΤΩΝ ΜΕΛΙΣΣΩΝ

15 Μετακινήσατε κάποια μελίσσια σας τουλάχιστον μία φορά για παραγωγή μελιού ή επικονίαση μέσα στο 2018;

○ **Ναι** ○ **Όχι** ○ **Δεν γνωρίζω**

α) Εάν ΝΑΙ, πόσες φορές κατά μέσο όρο? **ΑΡΙΘΜΟΣ** ____________

β)Ποια ήταν η μέση απόσταση των μετακινήσεων?

○ **15-50 χλμ** ○ **50-100 χλμ** ○ **100-250 χλμ** ○ **250-500 χλμ** ○ **>500 χλμ**

16 Πόσα τελάρα αντικαταστήσατε με φύλλα κηρήθρας ανά μελίσσι κατά μέσο όρο στο 2018 ?

○ **0%** ○ **1-30%** ○ **30-50%** ○ **>50%**

17 Είχε η πλειοψηφία των μελισσιών σας σημαντική παραγωγή σε ένα ή περισσότερα από τα παρακάτω φυτά; Παρακαλούμε επιλέξτε μία απάντηση για κάθε φυτό:

α) **Εσπεριδοειδή** ○ Ναι ○ Όχι ○ Δεν γνωρίζω

β) **Ελαιοκράμβη** ○ Ναι ○ Όχι ○ Δεν γνωρίζω

γ) **Καλαμπόκι** ○ Ναι ○ Όχι ○ Δεν γνωρίζω

δ) **Ηλίανθος** ○ Ναι ○ Όχι ○ Δεν γνωρίζω

ε) **Σουσούρα** ○ Ναι ○ Όχι ○ Δεν γνωρίζω

στ) **Πεύκο ή Έλατο** ○ Ναι ○ Όχι ○ Δεν γνωρίζω

ζ) **Θυμάρι** ○ Ναι ○ Όχι ○ Δεν γνωρίζω

η) **Βαμβάκι** ○ Ναι ○ Όχι ○ Δεν γνωρίζω

θ)**Λαδανιά** ○ Ναι ○ Όχι ○ Δεν γνωρίζω

ι) **Άλλα** : ___________

ΒΑΡΡΟΑ

18 Ελέγχατε τα μελίσσια σας για το ποσοστό από βαρρόα κατά την περίοδο από τον Απρίλη του 2018 μέχρι τον Απρίλη του 2019;

○ **Ναι** ○ **Όχι** ○ **Δεν γνωρίζω/δεν ισχύει**

19 Υποβάλατε σε θεραπεία τα μελίσσια σας κατά του βαρρόα κατά την περίοδο από τον Απρίλη του 2018 μέχρι τον Απρίλη του 2019;

○ **Ναι** ○ **Όχι** ○ **Δεν γνωρίζω/δεν ισχύει**

	2018									2019			
**20** Παρακαλούμε να σημειώσετε τους μήνες, κατά τους οποίους μετρούσατε τα παραγωγικά σας μελίσσια για το ποσοστό του βαρρόα **ΚΑΙ** σημειώστε επίσης πότε **ΞΕΚΙΝΗΣΑΤΕ** θεραπεία ή κάποια πρακτική ενάντια στο βαρρόα κατά την περίοδο από τον Απρίλη του 2018 μέχρι τον Απρίλη του 2019	**Απρίλιος**	**Μάιος**	**Ιούνιος**	**Ιούλιος**	**Αύγουστος**	**Σεπτέμβριος**	**Οκτώβριος**	**Νοέμβριος**	**Δεκέμβριος**	**Ιανουάριος**	**Φεβρουάριος**	**Μάρτιος**	**Απρίλιος**
Μετρήσεις του επιπέδου προσβολής από το βαρρόα	☐	☐	☐	☐	☐	☐	☐	☐	☐	☐	☐	☐	☐
Αφαίρεση κηφηνογόνου	☐	☐	☐	☐	☐	☐	☐	☐	☐	☐	☐	☐	☐
Υπερθερμία (Θεραπεία υπερθέρμανσης του γόνου/των μελισσών)	☐	☐	☐	☐	☐	☐	☐	☐	☐	☐	☐	☐	☐
Άλλες τεχνικές (π. Χ. Ολική αφαίρεση γόνου, περιορισμός βασίλισσας, κλπ)	☐	☐	☐	☐	☐	☐	☐	☐	☐	☐	☐	☐	☐
Φορμικό Οξύ - βραχυπρόθεσμα	☐	☐	☐	☐	☐	☐	☐	☐	☐	☐	☐	☐	☐
Φορμικό Οξύ - μακροπρόθεσμα	☐	☐	☐	☐	☐	☐	☐	☐	☐	☐	☐	☐	☐
Γαλακτικό οξύ	☐	☐	☐	☐	☐	☐	☐	☐	☐	☐	☐	☐	☐
Οξαλικό οξύ - διαβροχή	☐	☐	☐	☐	☐	☐	☐	☐	☐	☐	☐	☐	☐
Οξαλικό οξύ - εξάχνωση	☐	☐	☐	☐	☐	☐	☐	☐	☐	☐	☐	☐	☐
Ταινίες οξαλικού με γλυκερίνη (ΤΑΚ-ΤΙΚ)	☐	☐	☐	☐	☐	☐	☐	☐	☐	☐	☐	☐	☐
Μίγματα οξαλικού με άλλα (Hiveclean/Bienenwohl/Varromed)	☐	☐	☐	☐	☐	☐	☐	☐	☐	☐	☐	☐	☐
Θυμόλη (π.χ. Apiguard, ApilifeVar)	☐	☐	☐	☐	☐	☐	☐	☐	☐	☐	☐	☐	☐
Tau-fluvalinate (π.χ. Apistan)	☐	☐	☐	☐	☐	☐	☐	☐	☐	☐	☐	☐	☐
Φλουμεθρίνη (π.χ.. Bayvarol)	☐	☐	☐	☐	☐	☐	☐	☐	☐	☐	☐	☐	☐
Amitraz σε ταινιές	☐	☐	☐	☐	☐	☐	☐	☐	☐	☐	☐	☐	☐
Amitraz (υποκαπνισμός/αεροζόλ)	☐	☐	☐	☐	☐	☐	☐	☐	☐	☐	☐	☐	☐
Coumaphos (π.χ. Perizin)	☐	☐	☐	☐	☐	☐	☐	☐	☐	☐	☐	☐	☐
Coumaphos (σε ταινίες π.χ. Checkmite+)	☐	☐	☐	☐	☐	☐	☐	☐	☐	☐	☐	☐	☐
Άλλο χημικό προϊόν	☐	☐	☐	☐	☐	☐	☐	☐	☐	☐	☐	☐	☐
Άλλη μέθοδος	☐	☐	☐	☐	☐	☐	☐	☐	☐	☐	☐	☐	☐

ΕΠΙΠΛΕΟΝ ΠΡΟΑΙΡΕΤΙΚΕΣ ΕΡΩΤΗΣΕΙΣ

21 Εάν κάνατε στα μελισσοκομεία σας επιπλέον τροφοδότηση με σιρόπι (διάλυμα ζάχαρης ή ινβερτοποιημένο σιρόπι ή βανίλια) το προηγούμενο φθινόπωρο για να προετοιμαστείτε για το χειμώνα, πόσα κιλά ζάχαρης (στερεή μορφή) χρησιμοποιήσατε κατά μέσο όρο σε κάθε παραγωγικό μελίσσι; **ΑΡΙΘΜΟΣ** ________________

22 Διατηρείτε τα μελίσσια σας σε περιοχή που δεν έχει ανιχνευτεί ακόμα το βαρρόα;

◯**Ναι** ◯**Όχι** ◯**Δεν γνωρίζω**

23 Έχετε παρατηρήσει μέλισσες με κατεστραμμένα/παραμορφωμένα φτερά στα μελισσοκομεία σας (κατά τη διάρκεια του καλοκαιριού); Η εμφάνιση τέτοιων φτερών δηλώνει την παρουσία του Ιού των Παραμορφωμένων Φτερών, ο οποίος μεταδίδεται με το βαρρόα.

◯**καθόλου** ◯**σε μικρό ποσοστό** ◯**σε μεγάλο ποσοστό** ◯**Δεν γνωρίζω**

24 Ποια από τα παρακάτω ισχύουν κυρίως για την μελισσοκομική σας πρακτική? :

α) Διάτρητες βάσεις και τον χειμώνα ◯**Ναι** ◯**Όχι** ◯**Δεν γνωρίζω**

β) Κυψέλες με μόνωση για τον χειμώνα(διπλά τοιχώματα) ◯**Ναι** ◯**Όχι** ◯**Δεν γνωρίζω**

γ) Κυψέλες φτιαγμένες από συνθετικό υλικό ◯**Ναι** ◯**Όχι** ◯**Δεν γνωρίζω**

δ) Πιστοποιημένη βιολογική μελισσοκομεία ◯**Ναι** ◯**Όχι** ◯**Δεν γνωρίζω**

ε) Βασίλισσες από γενετικό υλικό ανθεκτικό στο βαρρόα ◯**Ναι** ◯**Όχι** ◯**Δεν γνωρίζω**

στ) Κηρήθρα με Μικρό μέγεθος κελιού του γόνου (5.1 mm ή λιγότερο) ◯**Ναι** ◯**Όχι** ◯**Δεν γνωρίζω**

ζ) Κηρήθρα που χτίζεται χωρίς φύλλο κηρήθρας ως βάση ◯**Ναι** ◯**Όχι** ◯**Δεν γνωρίζω**

η) Αγορά κεριού και από άλλους ◯**Ναι** ◯**Όχι** ◯**Δεν γνωρίζω**

θ) Συνθετικές κηρήθρες στον εμβρυϊκό θάλαμο ◯**Ναι** ◯**Όχι** ◯**Δεν γνωρίζω**

25 Παρατηρήσατε τη Vespa velutina να ψάχνει μέλισσες για να τραφεί στο μελισσοκομείο/μελισσοκομεία σας; ◯**Ναι** ◯**Όχι** ◯**Δεν γνωρίζω**

26 Πόσο εκτιμάτε (σε ευρώ) τα έξοδα ανά κυψέλη για τη θεραπεία του βαρρόα κατά την περίοδο Απρίλιος 2018 – Απρίλιος 2019; **ΑΡΙΘΜΟΣ** _______________

Θα θέλατε να προσθέσετε κάτι άλλο?

____
____
____
____

Σας ευχαριστούμε πολύ για τη συμμετοχή σας!

Αποθηκεύστε και αποστείλετε στο e-mail: beemonitorgr@gmail.com

ΠΛΗΡΟΦΟΡΙΕΣ – ΟΔΗΓΙΕΣ ΓΙΑ ΤΟ ΕΡΩΤΗΜΑΤΟΛΟΓΙΟ ΑΥΤΟ

Τι είναι το COLOSS;

Είναι μία Διεθνής Επιστημονική Ένωση που αποτελείται από 1019 μέλη από 95 χώρες που έχει στόχο την βελτίωση των συνθηκών ζωής των μελισσών.

Ποιοι είμαστε εμείς;

Είμαστε ερευνητές/μέλη του COLOSS που συμμετέχουμε σε μία έρευνα για την παρακολούθηση απωλειών μελισσών στην Ελλάδα και το πως αυτές οι απώλειες σχετίζονται με παράγοντες όπως πχ το βαρρόα. Αντίστοιχη έρευνα γίνεται και σε πολλές άλλες χώρες.

Τι μπορείτε να κάνετε;

Η παρακολούθηση των μελισσών γίνεται μέσω ενός ερωτηματολογίου που για να συμπληρωθεί απαιτούνται μόνο λίγα λεπτά. Θα σας παρακαλούσαμε να το συμπληρώσετε. Το ερωτηματολόγιο, αν το επιθυμείτε, είναι τελείως ανώνυμο.

Τι περιέχει το ερωτηματολόγιο ;

Η βασική ερώτηση είναι πόσα μελίσσια χάθηκαν μέσα στον τελευταίο χειμώνα. Επιπλέον έχει κάποιες ερωτήσεις που αφορούν τις αιτίες των απωλειών των μελισσιών.

Γιατί να ενδιαφερθώ ;

Αν είστε μελισσοκόμος χρόνια γνωρίζετε ήδη αρκετά πράγματα και θεωρείστε επαγγελματίας. Ερευνητές σε παγκόσμια κλίμακα αφιέρωσαν χρόνο για να ανακαλύψουν αυτά που σήμερα θεωρούμε δεδομένα. Υπάρχει περιθώριο για μεγάλη ακόμα πρόοδο στο πεδίο της μελισσοκομίας. Η έρευνα χρειάζεται τους μελισσοκόμους ώστε να δείξουν τον δρόμο και τα προβλήματα που υπάρχουν.

Ελληνική Ομάδα παρακολούθησης Απωλειών Μελισσιών

Σολεν Παταλανο, Δρ. COLOSS Συντονιστής
Βιολόγος, Ινστιτούτο Φλέμινγκ, Βάρη
Βαΐα Βουκάτα Φοιτήτρια Γεωπονικό Πανεπιστήμιο Αθηνών

Φανή Χατζήνα Δρ. Βιολόγος, Ερευνήτρια Α΄
Τμήμα Μελισσοκομίας ΕΛΓΟ «ΔΗΜΗΤΡΑ»
Φίλιππος Βαρδάκας, Εξωτερικός Συνεργάτης

www.coloss.org

Ερωτηματολόγιο για τις απώλειες των μελισσών 2019-2020

Αποστείλετε αυτό το ερωτηματολόγιο στο e-mail: beemonitorgr@gmail.com

Έως τις 1 ΙΟΥΛΙΟΥ 2020

Για περισσότερες πληροφορίες επικοινωνήστε μαζί μας! Monitoring Greece - Beekeepers

ΠΡΟΣΩΠΙΚΕΣ ΠΛΗΡΟΦΟΡΙΕΣ

Οι πληροφορίες ονόματος και διεύθυνσης δεν είναι υποχρεωτικές. Εάν τις δώσετε, θα κρατηθούν με μυστικότητα και δεν παρέχονται σε τρίτους για την προστασία του απορρήτου σας.

Όνομα	______________________________
Επίθετο	______________________________
Διεύθυνση	______________________________
Πόλη	______________________________
ΤΚ	______________________________
Email	______________________________

Οι ερωτήσεις σε κύκλο (❶, ❷, ❸...) είναι υποχρεωτικές

Οι ερωτήσεις σε Τετράγωνο (10, 11, 12...) είναι προαιρετικές

ΤΟΠΟΣ

❶ Για να γνωρίζουμε καλύτερα σε ποια περιοχή ήταν τα περισσότερα μελίσσια σας τον χειμώνα* που πέρασε, πέστε μας

α) Ποιος είναι ο ΝΟΜΟΣ που βρίσκεται το κυρίως μελισσοκομείο σας ; ____________

β) Το όνομα της κοντινότερης πόλης ______________

γ) Τι είδος περιοχής ήταν; ☐**Δάσος** ☐**Λιβάδι** ☐**Πόλη** ☐**Άλλο**:______________

*Χειμώνας θεωρείται η περίοδος μετά τις τελευταίες προετοιμασίες για το κρύο μέχρι την έναρξη της νέας περιόδου συλλογής τροφής

ΜΕΛΙΣΣΟΚΟΜΕΙΑ (ΟΜΑΔΕΣ ΚΥΨΕΛΩΝ)

❷ Πόσα μελισσοκομεία έχετε; **ΑΡΙΘΜΟΣ**: _______

❸ Εάν έχετε πάνω από ένα μελισσοκομείο, είναι η απόσταση μεταξύ όλων των μελισσοκομείων σας μέχρι 15 χλμ; Εάν έχετε μόνο ένα μελισσοκομείο, παρακαλώ να απαντήσετε Ναι.

☐ **Ναι** ☐ **Όχι** ☐ **Δεν γνωρίζω**

ΠΑΡΑΓΩΓΙΚΑ ΜΕΛΙΣΣΙΑ

4 Πόσα **παραγωγικά μελίσσια*** είχατε πριν το χειμώνα του 2019-2020; **ΑΡΙΘΜΟΣ:** ________

***Παραγωγικά μελίσσια θεωρούνται τα μελίσσια που είναι αρκετά υγιή για να θεωρήσετε ότι θα ξεχειμωνιάσουν**

Στις παρακάτω ερωτήσεις πρέπει να απαντήσετε για τις απώλειες των μελισσιών σας.
Θεωρείστε ΑΠΩΛΕΙΑ ένα μελίσσι που :
α) είναι ζωντανό αλλά έχει σοβαρά προβλήματα βασίλισσας (π.χ. έχει χαθεί η βασίλισσα ή είναι αρρενοτόκο ή δεν μπορέσατε να κάνετε κάτι)
β) χάθηκε λόγω φυσικών καταστροφών (Χιόνια, πλημμύρες, αρκούδες, κλοπές),
γ) ήταν ολόκληρο νεκρό ή έχει μείνει με μερικές εκατοντάδες μέλισσες μόνο,

ΑΠΩΛΕΙΕΣ ΜΕΛΙΣΣΙΩΝ

Από τα **παραγωγικά σας μελίσσια**:

5 Πόσα μελίσσια **χάθηκαν** επειδή είχαν σοβαρά **προβλήματα βασιλισσών** κατά τη διάρκεια του χειμώνα; (π.χ. έχει χαθεί η βασίλισσα ή είναι αρρενοτόκο ή δεν μπορέσατε να κάνετε κάτι).
Εάν δεν υπήρχαν προβλήματα παρακαλούμε να απαντήσετε **0**. **ΑΡΙΘΜΟΣ :** ________

6 Πόσα μελίσσια **χάθηκαν**, λόγω **φυσικών καταστροφών**; (Χιόνια, πλημμύρες, αρκούδες, κλοπές άλλα…) **ΑΡΙΘΜΟΣ :** ________

7 Πόσα μελίσσια **χάθηκαν**, λόγω **θανάτων μελισσών ή κενών κυψελών**; (ήταν ολόκληρο νεκρό ή έχει μείνει με μερικές εκατοντάδες μέλισσες) **ΑΡΙΘΜΟΣ :** ________

8 Πόσα από τα νεκρά μελίσσια (από αυτά που απαντήσατε στην Ερώτηση 7) ή τις άδειες κυψέλες είχαν:

α)**Πολλές νεκρές μέλισσες** μέσα ή μπροστά από την κυψέλη **ΑΡΙΘΜΟΣ :** ________
β)**Λίγες ή καθόλου νεκρές μέλισσες** μέσα ή μπροστά από την κενή κυψέλη **ΑΡΙΘΜΟΣ :** ________
γ) Νεκρές εργάτριες μέσα στα κελιά **από πείνα** **ΑΡΙΘΜΟΣ :** ________
δ) **Νεκρές εργάτριες μέσα στα κελιά αλλά με επαρκή τροφή** στην κυψέλη **ΑΡΙΘΜΟΣ :** ________
ε) κανένα από τα παραπάνω ή **άγνωστες αιτίες** **ΑΡΙΘΜΟΣ :** ________

9 Πόσα μελίσσια από αυτά που **επέζησαν (είχαν βασίλισσα),** ήταν **αδύναμα μετά το χειμώνα;** **ΑΡΙΘΜΟΣ :** ________

ΕΞΕΛΙΞΗ ΤΩΝ ΜΕΛΙΣΣΩΝ

10 Πόσα παραγωγικά μελίσσια είχατε την α) **άνοιξη*** του 2019 (πέρυσι); ________
β) άνοιξη του 2020 (φέτος); ________

***Άνοιξη** : αρχή της περιόδου συλλογής τροφής

11 Πόσα από τα μελίσσια που επέζησαν τον χειμώνα είχαν νέα βασίλισσα (του 2019);
ΑΡΙΘΜΟΣ : ________

12 Τυχόν προβλήματα στις βασίλισσας που παρατηρήσατε τον άνοιξη και το καλοκαίρι του 2019 ήταν συγκριτικά με αυτά που συνήθως έχετε:

☐ **Περισσότερα** ☐ **Τα ίδια** ☐ **Λιγότερα** ☐ **Δεν γνωρίζω**

13 Κατά τη διάρκεια του χειμώνα, τα μελίσσια με τις παλιές βασίλισσες (του 2019) σε σύγκριση με τα μελίσσια με τις νέες βασίλισσες (του 2020) επέζησαν:

☐ **Καλύτερα** ☐ **Το ίδιο** ☐ **Χειρότερα** ☐ **Δεν γνωρίζω**

14 Πόσα από τα μελίσσια που <u>επέζησαν</u> είχαν μεγάλες ποσότητες περιττωμάτων μέσα στην κυψέλη;

ΑΡΙΘΜΟΣ :________

ΜΕΤΑΚΙΝΗΣΗ ΚΑΙ ΠΕΡΙΒΑΛΛΟΝ ΤΩΝ ΜΕΛΙΣΣΩΝ

15 Μετακινήσατε κάποια μελίσσια σας τουλάχιστον μία φορά για παραγωγή μελιού ή επικονίαση μέσα στο 2019;

☐ **Ναι** ☐ **Όχι** ☐ **Δεν γνωρίζω**

α) Εάν ΝΑΙ, πόσες φορές κατά μέσο όρο; **ΑΡΙΘΜΟΣ** :________

β) Ποια ήταν η μέση απόσταση των μετακινήσεων;

☐ **15-50 χλμ** ☐ **50-100 χλμ** ☐ **100-250 χλμ** ☐ **250-500 χλμ** ☐ **>500 χλμ**

16 Πόσα τελάρα αντικαταστήσατε με φύλλα κηρήθρας ανά μελίσσι κατά μέσο όρο στο 2019 ?

☐ **0%** ☐ **1-30%** ☐ **30-50%** ☐ **>50%**

17 Είχε η πλειοψηφία των μελισσιών σας σημαντική παραγωγή σε ένα ή περισσότερα από τα παρακάτω φυτά; Παρακαλούμε επιλέξτε μία απάντηση για κάθε φυτό:

α) **Εσπεριδοειδή** ☐ Ναι ☐ Όχι ☐ Δεν γνωρίζω

β) **Ελαιοκράμβη** ☐ Ναι ☐ Όχι ☐ Δεν γνωρίζω

γ) **Καλαμπόκι** ☐ Ναι ☐ Όχι ☐ Δεν γνωρίζω

δ) **Ηλίανθος** ☐ Ναι ☐ Όχι ☐ Δεν γνωρίζω

ε) **Σουσούρα** ☐ Ναι ☐ Όχι ☐ Δεν γνωρίζω

στ) **Πεύκο ή Έλατο** ☐ Ναι ☐ Όχι ☐ Δεν γνωρίζω

ζ) **Θυμάρι** ☐ Ναι ☐ Όχι ☐ Δεν γνωρίζω

η) **Βαμβάκι** ☐ Ναι ☐ Όχι ☐ Δεν γνωρίζω

θ) **Λαδανιά** ☐ Ναι ☐ Όχι ☐ Δεν γνωρίζω

ι) **Άλλα** : ___________

ΒΑΡΡΟΑ

18 Ελέγχατε τα μελίσσια σας για το ποσοστό από βαρρόα κατά την περίοδο από τον Απρίλη 2019 μέχρι τον Μάρτιο 2020; ☐ **Ναι** ☐ **Όχι** ☐ **Δεν γνωρίζω/δεν ισχύει**

19 Υποβάλατε σε θεραπεία τα μελίσσια σας κατά του βαρρόα κατά την περίοδο από τον Απρίλη 2019 μέχρι τον Μάρτιο 2020; ☐ **Ναι** ☐ **Όχι** ☐ **Δεν γνωρίζω/δεν ισχύει**

20 Παρακαλούμε να σημειώσετε τους μήνες, κατά τους οποίους μετρούσατε τα παραγωγικά σας μελίσσια για το ποσοστό του βαρρόα **ΚΑΙ** σημειώστε επίσης πότε **ΞΕΚΙΝΗΣΑΤΕ** θεραπεία ή κάποια πρακτική ενάντια στο βαρρόα κατά την περίοδο από τον Απρίλη 2019 μέχρι τον Μάρτιο 2020:

	**2019**									**2020**		
	**Απρίλιος**	**Μάιος**	**Ιούνιος**	**Ιούλιος**	**Αύγουστος**	**Σεπτέμβριος**	**Οκτώβριος**	**Νοέμβριος**	**Δεκέμβριος**	**Ιανουάριος**	**Φεβρουάριος**	**Μάρτιος**
Μετρήσεις του επιπέδου προσβολής από το βαρρόα												
Αφαίρεση κηφηνογόνου												
Υπερθερμία (Θεραπεία υπερθέρμανσης του γόνου/των μελισσών)												
Άλλες τεχνικές (π. Χ. Ολική αφαίρεση γόνου, περιορισμός βασίλισσας, κλπ)												
Φορμικό Οξύ - βραχυπρόθεσμα												
Φορμικό Οξύ - μακροπρόθεσμα												
Γαλακτικό οξύ												
Οξαλικό οξύ - διαβροχή												
Οξαλικό οξύ - εξάχνωση												
Μίγματα οξαλικού με άλλα (Hiveclean/Bienenwohl/Varromed)												
Ταινίες οξαλικού με γλυκερίνη												
ΤΑΚ-ΤΙΚ												
Θυμόλη (π.χ. Apiguard, ApilifeVar)												
Tau-fluvalinate (π.χ. Apistan)												
Φλουμεθρίνη (π.χ.. Bayvarol)												
Amitraz σε ταινιές												
Amitraz (υποκαπνισμός/αεροζόλ)												
Coumaphos (π.χ. Perizin)												
Coumaphos (σε ταινίες π.χ. Checkmite+)												
Άλλο χημικό προϊόν												
Άλλη μέθοδος												

ΕΠΙΠΛΕΟΝ ΠΡΟΑΙΡΕΤΙΚΕΣ ΕΡΩΤΗΣΕΙΣ

21 Εάν κάνατε στα μελισσοκομεία σας επιπλέον τροφοδότηση με σιρόπι (διάλυμα ζάχαρης ή ινβερτοποιημένο σιρόπι ή βανίλια) το προηγούμενο φθινόπωρο για να προετοιμαστείτε για το χειμώνα, πόσα κιλά ζάχαρης (στερεή μορφή) χρησιμοποιήσατε κατά μέσο όρο σε κάθε παραγωγικό μελίσσι; **ΑΡΙΘΜΟΣ :**________

22 Διατηρείτε τα μελίσσια σας σε περιοχή που δεν έχει ανιχνευτεί ακόμα το βαρρόα;

☐ **Ναι** ☐ **Όχι** ☐ **Δεν γνωρίζω**

23 Έχετε παρατηρήσει μέλισσες με κατεστραμμένα/παραμορφωμένα φτερά στα μελισσοκομεία σας (κατά τη διάρκεια του καλοκαιριού); Η εμφάνιση τέτοιων φτερών δηλώνει την παρουσία του Ιού των Παραμορφωμένων Φτερών, ο οποίος μεταδίδεται με το βαρρόα.

☐ **καθόλου** ☐ **σε μικρό ποσοστό** ☐ **σε μεγάλο ποσοστό** ☐ **Δεν γνωρίζω**

24 Ποια από τα παρακάτω ισχύουν κυρίως για την μελισσοκομική σας πρακτική? :

α) Διάτρητες βάσεις και τον χειμώνα	☐ **Ναι** ☐ **Όχι** ☐ **Δεν γνωρίζω**
β) Κυψέλες με μόνωση για τον χειμώνα(διπλά τοιχώματα)	☐ **Ναι** ☐ **Όχι** ☐ **Δεν γνωρίζω**
γ) Κυψέλες φτιαγμένες από συνθετικό υλικό	☐ **Ναι** ☐ **Όχι** ☐ **Δεν γνωρίζω**
δ) Πιστοποιημένη βιολογική μελισσοκομεία	☐ **Ναι** ☐ **Όχι** ☐ **Δεν γνωρίζω**
ε) Βασίλισσες από γενετικό υλικό ανθεκτικό στο βαρρόα	☐ **Ναι** ☐ **Όχι** ☐ **Δεν γνωρίζω**
στ) Κηρήθρα με Μικρό μέγεθος κελιού του γόνου (5.1mm ή λιγότερο)	☐ **Ναι** ☐ **Όχι** ☐ **Δεν γνωρίζω**
ζ) Κηρήθρα που χτίζεται χωρίς φύλλο κηρήθρας ως βάση	☐ **Ναι** ☐ **Όχι** ☐ **Δεν γνωρίζω**
η) Αγορά κεριού και από άλλους	☐ **Ναι** ☐ **Όχι** ☐ **Δεν γνωρίζω**
θ) Συνθετικές κηρήθρες στον εμβρυϊκό θάλαμο	☐ **Ναι** ☐ **Όχι** ☐ **Δεν γνωρίζω**

25 Παρατηρήσατε τη Vespa velutina να ψάχνει μέλισσες για να τραφεί στο μελισσοκομείο/ μελισσοκομεία σας; ☐ **Ναι** ☐ **Όχι** ☐ **Δεν γνωρίζω**

26 Πόσο εκτιμάτε (σε ευρώ) τα έξοδα ανά κυψέλη για τη θεραπεία του βαρρόα κατά την περίοδο Απρίλιος 2019 – Μάρτιο 2020; **ΑΡΙΘΜΟΣ :**________

Θα θέλατε να προσθέσετε κάτι άλλο?

____
____
____
____

Σας ευχαριστούμε πολύ για τη συμμετοχή σας!

Αποθηκεύστε και αποστείλετε στο e- mail: beemonitorgr@gmail.com

ΠΛΗΡΟΦΟΡΙΕΣ – ΟΔΗΓΙΕΣ ΓΙΑ ΤΟ ΕΡΩΤΗΜΑΤΟΛΟΓΙΟ ΑΥΤΟ

Τι είναι το COLOSS;

Είναι μία Διεθνής Επιστημονική Ένωση που αποτελείται από 1019 μέλη από 95 χώρες που έχει στόχο την βελτίωση των συνθηκών ζωής των μελισσών.

Ποιοι είμαστε εμείς;

Είμαστε ερευνητές/μέλη του COLOSS που συμμετέχουμε σε μία έρευνα για την παρακολούθηση απωλειών μελισσών στην Ελλάδα και το πως αυτές οι απώλειες σχετίζονται με παράγοντες όπως πχ το βαρρόα. Αντίστοιχη έρευνα γίνεται και σε πολλές άλλες χώρες.

Τι μπορείτε να κάνετε;

Η παρακολούθηση των μελισσών γίνεται μέσω ενός ερωτηματολογίου που για να συμπληρωθεί απαιτούνται μόνο λίγα λεπτά. Θα σας παρακαλούσαμε να το συμπληρώσετε. Το ερωτηματολόγιο, αν το επιθυμείτε, είναι τελείως ανώνυμο.

Τι περιέχει το ερωτηματολόγιο ;

Η βασική ερώτηση είναι πόσα μελίσσια χάθηκαν μέσα στον τελευταίο χειμώνα. Επιπλέον έχει κάποιες ερωτήσεις που αφορούν τις αιτίες των απωλειών των μελισσιών.

Γιατί να ενδιαφερθώ ;

Αν είστε μελισσοκόμος χρόνια γνωρίζετε ήδη αρκετά πράγματα και θεωρείστε επαγγελματίας. Ερευνητές σε παγκόσμια κλίμακα αφιέρωσαν χρόνο για να ανακαλύψουν αυτά που σήμερα θεωρούμε δεδομένα. Υπάρχει περιθώριο για μεγάλη ακόμα πρόοδο στο πεδίο της μελισσοκομίας. Η έρευνα χρειάζεται τους μελισσοκόμους ώστε να δείξουν τον δρόμο και τα προβλήματα που υπάρχουν.

Ελληνική Ομάδα παρακολούθησης Απωλειών Μελισσιών

Φανή Χατζήνα Δρ. Βιολόγος, Ερευνήτρια Α΄
Τμήμα Μελισσοκομίας ΕΛΓΟ «ΔΗΜΗΤΡΑ»
Σολεν Παταλανο, Δρ. COLOSS Συντονιστής
Βιολόγος, Ινστιτούτο Φλέμινγκ, Βάρη
Φίλιππος Βαρδάκας, Εξωτερικός Συνεργάτης

Ερωτηματολόγιο Ελλάδα 2020-2021

Αγαπητέ μελισσοκόμε,
Παρακάτω θα βρείτε την ανανεωμένη και απλοποιημένη έκδοση του ετήσιου μας ερωτηματολογίου. Σας παρακαλούμε να το συμπληρώσετε και στείλετε **μέχρι την 1η Ιουλίου 2021**, κι ας το έχετε κάνει στο παρελθόν. Για περισσότερες πληροφορίες και τα περσινά αποτελέσματα, επισκεφτείτε μας στο: www.facebook.com/beemonitorgr

Σας ευχαριστούμε!

Σημειώστε ότι οι ερωτήσεις με ● είναι **υποχρεωτικές.**

ΣΤΟΙΧΕΙΑ ΜΕΛΙΣΣΟΚΟΜΟΥ

1. Στοιχεία επικοινωνίας

Οι πληροφορίες ονόματος και διεύθυνσης δεν είναι υποχρεωτικές. Εάν τις δώσετε, θα κρατηθούν με μυστικότητα και δεν παρέχονται σε τρίτους για την προστασία του απορρήτου σας.

Ονοματεπώνυμο

e-mail

● 2. Ποιος είναι ο Νομός που βρίσκεται το κυρίως μελισσοκομείο σας;

Νομός

● 3. Για να γνωρίζουμε καλύτερα σε ποια περιοχή ήταν τα περισσότερα μελίσσια σας τον χειμώνα που πέρασε, πέστε μας το όνομα της κοντινότερης πόλης:

Πόλη

● 4. Πόσα μελισσοκομεία έχετε;

Μελισσοκομεία εννοούνται οι ομάδες κυψελών και όχι το σύνολο των κυψελών σας.

● 5. Πόσες παραγωγικές κυψέλες είχατε πριν το χειμώνα του 2020-2021;

Παραγωγικές κυψέλες θεωρούνται αυτές που είναι αρκετά υγιείς για να ξεχειμωνιάσουν.

ΧΕΙΜΕΡΙΝΕΣ ΑΠΩΛΕΙΕΣ ΜΕΛΙΣΣΩΝ

● 6. Από τις παραγωγικές σας κυψέλες μετά το χειμώνα, πόσες είχαν:

Εάν δεν υπήρχαν προβλήματα παρακαλούμε να απαντήσετε 0.

α) Απώλειες με σοβαρά **προβλήματα βασιλισσών;**

β) Απώλειες λόγω **φυσικών καταστροφών;**

γ) Απώλειες λόγω **θανάτων μελισσών;**

Σύνολο:

7. Από τις κυψέλες που είχαν απώλειες λόγω **θανάτων μελισσών** (ερώτηση "**6γ**"), πόσες είχαν: *Εάν δεν υπήρχαν προβλήματα παρακαλούμε να απαντήσετε 0.*

α) **πολλές νεκρές** μέλισσες μέσα ή μπροστά από την κυψέλη;

β) **λίγες ή καθόλου νεκρές μέλισσες** σε κενή κυψέλη;

γ) νεκρές εργάτριες μέσα στα κελιά από **πείνα**;

δ) Νεκρές εργάτριες μέσα στα κελιά **αλλά με τροφή**;

ε) κανένα από τα παραπάνω ή **άγνωστες αιτίες**;

Σύνολο:

Το "Σύνολο" πρέπει να είναι το ίδιο με το (6γ) νούμερο χαμένων κυψελών.

ΒΑΣΙΛΙΣΣΕΣ

● 8. Πόσες από τις κυψέλες που επέζησαν τον χειμώνα είχαν νέα βασίλισσα (του2020);

9. Σε ποιο βαθμό παρατηρήσατε τυχόν προβλήματα στις βασίλισσες την άνοιξη και το καλοκαίρι του 2020 συγκριτικά με άλλες χρονιές; *Επιλέξτε **μια** από τις παρακάτω απαντήσεις.*

☐ Περισσότερα ☐ Λιγότερα ☐ Τα ίδια

☐ Καμία απάντηση ☐ Δεν γνωρίζω

10. Κατά τη διάρκεια του χειμώνα, οι κυψέλες με παλιές βασίλισσες (του 2020) σε σύγκριση με τις κυψέλες με νέες βασίλισσες (του 2021) επέζησαν:

*Επιλέξτε **μια** από τις παρακάτω απαντήσεις.*

☐ Καλύτερα ☐ Χειρότερα ☐ Το ίδιο

☐ ☐

ΠΕΡΙΒΑΛΛΟΝ - ΜΕΤΑΚΙΝΗΣΗ

● 11. Η περιοχή που πέρασαν οι κυψέλες σας το χειμώνα ήταν:

***Χειμώνας** θεωρείται η περίοδος μετά τις τελευταίες προετοιμασίες για το κρύο μέχρι την έναρξη της νέας περιόδου συλλογής τροφής. Επιλέξτε **μια** από τις παρακάτω απαντήσεις.*

☐ Δάσος ☐ Πόλη ☐ Λιβάδι

☐ Καλλιέργειες ☐ Κανένα από παραπάνω

● 12. Μετακινήσατε κάποια μελίσσια σας τουλάχιστον μία φορά για παραγωγή μελιού ή επικονίαση μέσα στο 2020;

☐ Ναι ☐ Όχι

Αν ναι, πόσες φορές; Πόσα συνολικά χλμ;

● 13. Έδωσε η πλειοψηφία των μελισσιών σας σημαντική παραγωγή σε ένα ή περισσότερα από τα παρακάτω φυτά;

	Ναι	Αβέβαιο	Όχι		Ναι	Αβέβαιο	Όχι
α) Εσπεριδοειδή	☐	☐	☐	ε) Σουσούρα	☐	☐	☐
β) Ελαιοκράμβη	☐	☐	☐	στ) Πεύκο/Έλατο	☐	☐	☐
γ) Καλαμπόκι	☐	☐	☐	ζ) Θυμάρι	☐	☐	☐
δ) Ηλίανθος	☐	☐	☐	η) Βαμβάκι	☐	☐	☐
θ) Λαδανιά	☐	☐	☐	Άλλο:			

ΒΑΡΡΟΑ

● 14. Ελέγχετε τις κυψέλες σας για βαρρόα;

☐ Ναι ☐ Όχι

Αν Ναι, ποιες μεθόδους χρησιμοποιείτε για τον έλεγχο του βαρρόα;

☐ Πλύση με Αλκοόλ
☐ Φυσικής πτώσης (ανοιχτό πάτωμα)
☐ Ζάχαρη άχνη / Σέϊκερ
☐ Οπτική επιθεώρηση κηφήνων
☐ Δείγμα σε εργαστήριο
☐ Τακτική επιθεώρηση μελισσών
☐ Άλλη μέθοδος:

● 15. Υποβάλατε σε θεραπεία τις μέλισσές σας κατά του βαρρόα;

☐ Ναι ☐ Όχι

16. Παρακαλούμε σημειώσετε τους μήνες κατά τους οποίους μετρήσατε το βαρρόα ΚΑΙ επίσης πότε ΞΕΚΙΝΗΣΑΤΕ θεραπεία ενάντια στο βαρρόα:

Εάν χρησιμοποιήσατε την ίδια μέθοδο για συνεχόμενους μήνες, σημειώστε μόνο τον πρώτο μήνα.

	2020									2021		
	Απρ	Μάι	Ιούν	Ιούλ	Αυγ	Σεπ	Οκτ	Νοε	Δεκ	Ιαν	Φεβ	Μαρ
Μετρήσεις του επιπέδου προσβολής από το βαρρόα	☐	☐	☐	☐	☐	☐	☐	☐	☐	☐	☐	☐
Αφαίρεση κηφηνογόνου	☐	☐	☐	☐	☐	☐	☐	☐	☐	☐	☐	☐
Υπερθερμία	☐	☐	☐	☐	☐	☐	☐	☐	☐	☐	☐	☐
Άλλες τεχνικές (περιορισμός βασίλισσας...)	☐	☐	☐	☐	☐	☐	☐	☐	☐	☐	☐	☐
Φορμικό Οξύ - βραχυπρόθεσμα	☐	☐	☐	☐	☐	☐	☐	☐	☐	☐	☐	☐
Φορμικό Οξύ - μακροπρόθεσμα	☐	☐	☐	☐	☐	☐	☐	☐	☐	☐	☐	☐
Γαλακτικό οξύ	☐	☐	☐	☐	☐	☐	☐	☐	☐	☐	☐	☐
Οξαλικό οξύ - διαβροχή	☐	☐	☐	☐	☐	☐	☐	☐	☐	☐	☐	☐
Οξαλικό οξύ - εξάχνωση	☐	☐	☐	☐	☐	☐	☐	☐	☐	☐	☐	☐
Μίγματα οξαλικού με άλλα (Hiveclean/Bienenwohl/Varromed)	☐	☐	☐	☐	☐	☐	☐	☐	☐	☐	☐	☐
Θυμόλη (π.χ. Apiguard, ApilifeVar)	☐	☐	☐	☐	☐	☐	☐	☐	☐	☐	☐	☐
Tau-fluvalinate (π.χ. Apistan)	☐	☐	☐	☐	☐	☐	☐	☐	☐	☐	☐	☐
Φλουμεθρίνη (π.χ. Bayvarol)	☐	☐	☐	☐	☐	☐	☐	☐	☐	☐	☐	☐
Amitraz σε ταινιές / TakTik	☐	☐	☐	☐	☐	☐	☐	☐	☐	☐	☐	☐
Amitraz (υποκαπνισμός/αεροζόλ)	☐	☐	☐	☐	☐	☐	☐	☐	☐	☐	☐	☐
Coumaphos (π.χ. Perizin)	☐	☐	☐	☐	☐	☐	☐	☐	☐	☐	☐	☐
Coumaphos (σε ταινίες π.χ. Checkmite+)	☐	☐	☐	☐	☐	☐	☐	☐	☐	☐	☐	☐
Άλλο χημικό προϊόν	☐	☐	☐	☐	☐	☐	☐	☐	☐	☐	☐	☐
Άλλη μέθοδος:	☐	☐	☐	☐	☐	☐	☐	☐	☐	☐	☐	☐

17. Έχετε παρατηρήσει μέλισσες με κατεστραμμένα / παραμορφωμένα φτερά στα μελισσοκομεία σας;

*Η εμφάνιση τέτοιων φτερών δηλώνει την παρουσία του Ιού των Παραμορφωμένων Φτερών, ο οποίος μεταδίδεται με το Βαρρόα. Επιλέξτε **μια** από τις παρακάτω απαντήσεις.*

☐ Καθόλου ☐ Σε μικρό ποσοστό ☐ Σε μεγάλο ποσοστό

☐ Δεν γνωρίζω ☐ Καμία απάντηση

18. Πόσο εκτιμάτε τα έξοδα ανά κυψέλη για τη θεραπεία του βαρρόα κατά την περίοδο Απρίλιος 2020 – Μάρτιο 2021;

€

ΜΕΛΙΣΣΟΚΟΜΙΚΕΣ ΠΡΑΚΤΙΚΕΣ

19. Ποια από τα παρακάτω ισχύουν κυρίως για την μελισσοκομική σας πρακτική;

	Ναι	Αβέβαιο	Όχι
α) Διάτρητες βάσεις και τον χειμώνα	☐	☐	☐
β) Κυψέλες με μόνωση για τον χειμώνα (διπλά τοιχώματα)	☐	☐	☐
γ) Κυψέλες φτιαγμένες από συνθετικό υλικό	☐	☐	☐
δ) Πιστοποιημένη βιολογική μελισσοκομία	☐	☐	☐
ε) Βασίλισσες από γενετικό υλικό ανθεκτικό στο βαρρόα	☐	☐	☐
στ) Κηρήθρα με μικρό μέγεθος κελιού του γόνου (5.1mm ή λιγότερο)	☐	☐	☐
ζ) Κηρήθρα που χτίζεται χωρίς φύλλο κηρήθρας ως βάση	☐	☐	☐
η) Αγορά κεριού και από άλλους	☐	☐	☐
θ) Συνθετικές κηρήθρες στον εμβρυϊκό θάλαμο	☐	☐	☐

20. Πόσα πλαίσια αντικαταστήσατε με φύλλα κηρήθρας ανά μελίσσι κατά μέσο όρο στο 2020; *Επιλέξτε μια από τις παρακάτω απαντήσεις.*

☐ 0% ☐ 1-30% ☐ 31-50%

☐ πάνω από 50% ☐ Καμία απάντηση

21. Εάν κάνατε επιπλέον τροφοδότηση με σιρόπι (διάλυμα ζάχαρης ή ινβερτοποιημένο σιρόπι ή βανίλια) για να προετοιμαστείτε για το χειμώνα, πόσα κιλά ζάχαρης (στερεή μορφή) χρησιμοποιήσατε κατά μέσο όρο σε κάθε παραγωγική κυψέλη;

kg

Στείλτε το ερωτηματολόγιο **πριν από την 1η Ιουλίου** στο:
ΙΝΣΤΙΤΟΥΤΑ ΑΓΡΟΤΙΚΗΣ ΕΡΕΥΝΑΣ
Τμήμα Μελισσοκομίας- ΕΛΓΟ – ΔΗΜΗΤΡΑ
Νέα Μουδανιά, 63 200 Ελλάδα

Ή μέσω email στο:
beemonitorgr@gmail.com

Ευχαριστούμε πολύ για τη συμμετοχή σας!
